# Tackling Glaucoma from within the Brain: An Unfortunate Interplay of BDNF and TrkB

**DOI:** 10.1371/journal.pone.0142067

**Published:** 2015-11-11

**Authors:** Eline Dekeyster, Emiel Geeraerts, Tom Buyens, Chris Van den Haute, Veerle Baekelandt, Lies De Groef, Manuel Salinas-Navarro, Lieve Moons

**Affiliations:** 1 Neural Circuit Development and Regeneration Research Group, Department of Biology, KU Leuven, Leuven, Belgium; 2 Neurobiology and Gene Therapy Research Group, Department of Neurosciences, KU Leuven, Leuven, Belgium; 3 Leuven Viral Vector Core, KU Leuven, Leuven, Belgium; NIH/NEI, UNITED STATES

## Abstract

According to the neurotrophin deprivation hypothesis, diminished retrograde delivery of neurotrophic support during an early stage of glaucoma pathogenesis is one of the main triggers that induce retinal ganglion cell (RGC) degeneration. Therefore, interfering with neurotrophic signaling seems an attractive strategy to achieve neuroprotection. Indeed, exogenous neurotrophin administration to the eye has been shown to reduce loss of RGCs in animal models of glaucoma; however, the neuroprotective effect was mostly insufficient for sustained RGC survival. We hypothesized that treatment at the level of neurotrophin-releasing brain areas might be beneficial, as signaling pathways activated by target-derived neurotrophins are suggested to differ from pathways that are initiated at the soma membrane. In our study, first, the spatiotemporal course of RGC degeneration was characterized in mice subjected to optic nerve crush (ONC) or laser induced ocular hypertension (OHT). Subsequently, the well-known neurotrophin brain-derived neurotrophic factor (BDNF) was chosen as the lead molecule, and the levels of BDNF and its high-affinity receptor, tropomyosin receptor kinase B (TrkB), were examined in the mouse retina and superior colliculus (SC) upon ONC and OHT. Both models differentially influenced BDNF and TrkB levels. Next, we aimed for RGC protection through viral vector-mediated upregulation of collicular BDNF, thought to boost the retrograde neurotrophin delivery. Although the previously reported temporary neuroprotective effect of intravitreally delivered recombinant BDNF was confirmed, viral vector-induced BDNF overexpression in the SC did not result in protection of the RGCs in the glaucoma models used. These findings most likely relate to decreased neurotrophin responsiveness upon vector-mediated BDNF overexpression. Our results highlight important insights concerning the complexity of neurotrophic factor treatments that should surely be considered in future neuroprotective strategies.

## Introduction

Glaucomatous optic neuropathies are characterized by degeneration of retinal ganglion cells (RGCs) and their axons, resulting in visual field defects[1–3]. The pathophysiological mechanisms underlying glaucomatous RGC death are not completely understood and the sole target for clinical intervention at present is increased intraocular pressure (IOP) or ocular hypertension (OHT)[1–3]. Unfortunately, in a significant number of patients, IOP lowering cannot prevent the RGCs from dying[4], encouraging researchers to search for alternative treatment strategies that aim for neuroprotection of the RGCs.

One of the early events during the pathogenesis of glaucoma is axonal dystrophy within the optic nerve, perturbing axonal transport of neurotrophins[5, 6]. The neurotrophin deprivation hypothesis proposes that diminished retrograde transport, resulting in deprivation of brain-derived neurotrophic support to the RGCs, is one of the triggers that induces glaucomatous retinal cell death[5, 6]. Based on this theory, various experimental strategies have sought to supply exogenous neurotrophins to curb glaucomatous neurodegeneration[6]. Among the neurotrophins linked to glaucoma, brain-derived neurotrophic factor (BDNF) stands out for its vital role in maintaining RGCs and its potent protective effect in different glaucoma models[6–14]. BDNF is synthesized locally in the retina by RGCs[15] and glia[16, 17], but is also produced in the RGC projection areas in the brain from where it is taken up by RGC terminals and retrogradely transported, as a neurotrophin-receptor complex, via their axons to the cell bodies[5]. BDNF is synthesized as pro-BDNF and can be subsequently cleaved to form mature BDNF (mBDNF)[18]. The yin and yang model, stating that pro-BDNF merely induces neuronal apoptosis while mBDNF promotes survival[19], has recently been questioned[20, 21]. The neurotrophic effects of BDNF are primarily mediated through binding to its high-affinity receptor, tropomyosin receptor kinase B (TrkB)[13]. TrkB exists in different splice variants, of which TrkB-full length (TrkB-FL) and TrkB-truncated from 1 (TrkB-T1), lacking the C-terminal intracellular kinase domain, are the most abundant in the central nervous system[22]. Binding of BDNF to TrkB-FL induces receptor dimerization and transphosphorylation, leading to activation of intracellular signaling pathways involved in cell viability[18]. Until now, the exact role of TrkB-T1 remains less clear[22]. TrkB-T1 was first defined as a dominant-negative receptor that heterodimerizes with TrkB-FL to indirectly inhibit BDNF survival signaling. However, more recently, TrkB-T1 has, for example, also been linked to a complex web of intracellular glial signaling pathways, as well as to regulation of extracellular BDNF concentrations through sequestration [22].

Although local administration of BDNF to the eye has repeatedly been shown to reduce loss of RGCs in various glaucoma models[6–14], the neuroprotective effect is mostly insufficient to support sustained RGC survival. Alternative to retinal administration, it might be beneficial to consider treatment at the level of extraretinal neurotrophin sources in the brain, as target-derived neurotrophins are likely to induce signaling pathways that differ from local neurotrophin signaling[18, 23–25]. Recently, this hypothesis was partially confirmed by combining intravitreal (ivt) injection with chronic delivery of BDNF into the visual brain centers, which resulted in prolonged and enhanced RGC survival in cats subjected to optic nerve trauma[7, 26].

The overall objective of this study was to advance the knowledge of the role of neurotrophin signaling between the eye and the brain in glaucoma pathology. Therefore, two different mouse models of experimental glaucoma were used: the optic nerve crush (ONC) model, defined by mechanical trauma to the RGC axons in the optic nerve[27–29], and a laser induced ocular hypertension (OHT) model, in which IOP is temporary elevated through laser photocoagulation (LP) of the perilimbal and episcleral veins[30, 31]. Although rather acute, both models share important features with human glaucoma, where the primary injury to RGCs is also axonal and possibly occurs through OHT-related mechanical forces stressing axons in the optic nerve head (ONH)[32]. After outlining the RGC degeneration profiles, the temporal patterns of BDNF and TrkB-FL/TrkB-T1 levels in the retina and superior colliculus (SC), the primary target for RGC axons in rodents[33], were visualized in both models of experimentally induced glaucoma. Furthermore, the previously reported neuroprotective effect of ivt recombinant BDNF administration was re-examined in the ONC model and, in addition, the effect of viral vector-mediated BDNF upregulation in the SC on RGC survival/death was investigated in both the ONC and the OHT model.

## Materials and Methods

### Animals and anesthetics

All experiments were performed on adult C56Bl/6J and CD-1 mice (10–12 weeks old at the start of the experiment), housed under a 12/12 h light/dark cycle with *ad libitum* access to food and water. The experiments were approved by the KU Leuven Animal Ethics Committee and followed the guidelines of the ARVO statement for the use of animals in ophthalmic and Vision Res.

Surgical procedures to induce experimental glaucoma and for viral vector or recombinant BDNF administration were performed under general anaesthesia, induced by intraperitoneal (i.p.) administration of ketamine (75 mg/kg body weight, Anesketin, Eurovet, Belgium) and medetomidin (1 mg/kg body weight, Domitor, Janssen Pharmaceutics, Belgium). Additionally, for glaucoma induction and intravitreal (ivt) injections, a droplet of local analgesic, oxybuprocaïne hydrochloride (0.4%, Unicaïne, Théa, Belgium) was applied to the eye. For intracollicular vector injections, the head region was topically analgesized by applying lidocaine hydrochloride (5%, Xylocaine, AstraZeneca, UK) to the scalp. After any surgical procedure, waking was encouraged by i.p. administration of atipamezole (1 mg/kg body weight, Antisedan, Pfizer, Belgium). During recovery, tobramycin eye ointment (Tobrex, Alcon, TX, USA) was applied to the cornea of both eyes to prevent desiccation and mice were placed on a heat pad to maintain body temperature.

### Experimental glaucoma induction

In both models, experimental glaucoma was induced in the left eye (*oculus sinister*, OS) while the right eye (*oculus dexter*, OD) served as an internal control.

Optic nerve crush (ONC) was performed in C57Bl/6J mice as described previously[34]. Briefly, a small temporal incision was made in the conjunctiva and by gently retracting the conjunctiva, the globe was rotated nasally. Subsequently, blunt dissection was used to create a space between the temporal lateral rectus muscle and the inferior oblique muscle to expose the optic nerve. The optic nerve was then crushed 1 mm behind the globe for 5 s using self-closing cross-action forceps (11262–30, Fine Science Tools, Germany) in order to provide a constant and consistent pressure of 120 gr/mm^2^. Before and after the ONC procedure, funduscopy was performed to ensure a normal retinal perfusion.

Ocular hypertension (OHT) was induced in CD-1 mice through laser photocoagulation (LP) of the ocular veins as described before[30, 31, 35]. Briefly, after dilating the pupil by a droplet of tropicamide (1%, Mydriacyl, Alcon, TX, USA), a 532 nm diode laser (Vitra, Quantel Medical, France) was employed to deliver approximately 70 laser spots (0.5 s exposure time and 0.3 W power intensity) to the perilimbal and episcleral veins. Before and immediately after LP, funduscopy was performed to account for ischemic effects. The intraocular pressure (IOP) was measured at a defined daily time point in awake animals, before and up to 7 d after LP, using a calibrated rebound tonometer (Tono-Lab, Icare, Finland).

### Intravitreal BDNF administration

Mice received a 2 μl ivt injection of BDNF (Peprotech Laboratories, UK), diluted at 1 mg/ml in 0.01 M phosphate buffered saline (PBS) containing 1% bovine serum albumin (BSA, PanReac Applichem, Germany) (BSA-PBS). Ivt injections were performed using a glass capillary with a 50–70 μm outer diameter, connected to a Hamilton syringe and a Micro4 Microsyringe controller (World Precision Instruments, FL, USA), at a rate of 500 nl/s. Control groups received an ivt injection of 2 μl vehicle (BSA-PBS). Two main treatment paradigms were used: (i) a single dose immediately after crush with sacrifice at 7 or 14 days post-injury (dpi), and (ii) repeated ivt injection at 0, 3, and 5 d with sacrifice at 7 dpi, or at 0, 5, and 10 d with sacrifice at 14 d post ONC.

### Intracollicular BDNF overexpression induced by viral vector technology

To optimize viral vector transduction in the mouse SC, the tropism and transduction efficiency of four rAAV serotypes (2/1, 2/7, 2/8, and 2/9) were evaluated. For more information, see supplementary methods described in S1 Text.

In order to enhance BDNF levels in the SC, a murine BDNF-expressing vector was developed. RNA was isolated from mouse tail tissue using TRIzol reagent (Invitrogen, CA, USA) and cDNA synthesis was carried out following standard procedures[36]. PCR amplification of the BDNF cDNA was performed with Phusion high fidelity DNA polymerase (New England Biolabs, UK) using forward primer 5’-TCTAGAGATGACCATCCTTTTCCTTAC-3’ and reverse primer 5’-ACTAGTCTATCTTCCCCTTTTAATGGTC-3’. Subsequently, mouse BDNF cDNA was cloned using the Zero Blunt TOPO PCR Cloning kit (Invitrogen, CA, USA) and the resulting plasmid was transformed into competent *Escherichia coli* cells (One shot, Invitrogen, CA, USA). Plasmids were isolated from positive clones by use of the PureLink Quick Plasmid Miniprep kit (Invitrogen, CA, USA) and PCR was performed with forward primer 5’-AAAAAAGGATCCGCCACCATGACCATCCTTTTCCTTACTATGG-3’ and reverse primer 5’-GCCCTTACTAGTCTATCTTCCCCTTTTAATGGTCAGT-3’. Purified PCR products were ligated in the rAAV transfer plasmids[37] through BamH1 and Spe1 restriction sites. The resulting BDNF-rAAV plasmids were transformed into Max Efficiency Stbl2 competent cells (Invitrogen, CA, USA). Plasmids were isolated from positive clones using the GenElute Plasmid Miniprep kit (Sigma-Aldrich, MO, USA) and the construct was sequence verified before viral vector production.

The adeno-associated viral vector serotype 2/1 containing the mouse BDNF gene under control of a general cytomegalovirus (CMV) promoter (rAAV2/1-CMV-BDNF) and the control enhanced green fluorescent protein (eGFP)-expressing vector (rAAV2/1-CMV-eGFP) were produced by the Viral Vector Core of KU Leuven. The vector production process has been described in detail by Van der Perren *et al*.[38].

To induce unilateral overexpression of BDNF in the SC, 1 μl rAAV2/1-CMV-BDNF was stereotactically delivered into the right SC at 3.16E11 GC/ml, using the Nanoject II micro-injector (Drummond Scientific Company, PA, USA). Control groups received 1 μl injection of rAAV2/1-CMV-eGFP vector at 3.25E11 GC/ml. Coordinates for mouse right SC were anteroposterior -3.3 mm and mediolateral +0.4 mm relative to Bregma, and dorsoventral -1.3 mm from the dura.

### Detection of BDNF and TrkB levels

#### Sample processing and protein concentration

Animals were anesthetized with sodium pentobarbital (200 mg/kg body weight, Nembutal, Ceva Sante Animale, France) prior to cervical dislocation. Retinas and SCi were dissected, snap-frozen, and homogenized in RIPA buffer (25 mM Tris-HCl, 150 mM NaCl, 1% NP-40, 1% sodium deoxycholate, 0.1% sodium dodecyl sulfate, pH 7.6) containing a protease and phosphatase inhibitor cocktail (Thermo scientific, MA, USA). Protein concentrations were determined with the Qubit Protein Assay (Molecular probes, Invitrogen, CA, USA) and samples were stored at -80°C.

#### Western blotting

Levels of mBDNF, TrkB-FL, and TrkB-T1 were detected in the retina and SC samples using western blotting. Equal amounts of the sample (retina: BDNF 120 μg, TrkB 50 μg; SC: BDNF 60 μg, TrkB 20 μg) were loaded onto 4–12% Bis-Tris polyacrylamide gels (BioRad Laboratories, CA, USA) and transferred to polyvinylidene difluoride (PVDF) membranes (BioRad Laboratories, CA, USA). The membrane was incubated for 2 h in 5% ECL Blocking Agent (GE Healthcare, UK) in 0.01 M tris buffered saline (TBS). Overnight incubation with primary antibody, polyclonal rabbit anti-BDNF (1:500, sc-546, Santa Cruz Biotechnology, TX, USA) or polyclonal rabbit anti-TrkB (1:1500, 07–225, Millipore, MA, USA), was followed by 45 min incubation with horseradish peroxidase (HRP)-conjugated goat anti-rabbit IgG (1:10,000, Dako, Denmark). Specific protein bands were visualized using the luminol-based enhanced chemiluminescence kit (SuperSignal West Dura, Thermo Scientific, MA, USA). In order to normalize detected amounts of mBDNF or TrkB to the total amount of protein loaded, LavaPurple (Gelcompany, CA, USA) total protein stain was performed prior to primary antibody incubation. Specific protein bands and LavaPurple staining were imaged with the ChemiDoc MP Imaging System (BioRad Laboratories, CA, USA) and optical density analyses were performed using Image Lab 4.1 software (BioRad Laboratories, CA, USA).

#### ELISA

Total BDNF levels, including both pro- and mature forms, were quantified based on standard sandwich enzyme-linked immunosorbent assay (ELISA) technology by means of the Mouse BDNF ELISA kit (KA0331, Abnova, Taiwan). Data analyses were performed with Skanit 3.1 software (Thermo Scientific, MA, USA).

### Immunohistochemistry

#### Tissue processing

Animals were anesthetized through i.p. administration of sodium pentobarbital (200 mg/kg body weight, Nembutal, Ceva Sante Animale, France) and transcardially perfused with 0.9% NaCl, followed by 4% phosphate buffered paraformaldehyde (PFA, Acros Organics, NJ, USA) for standard immunohistochemistry (IHC). Following perfusion, eyes were enucleated and fixed for 1 h in 4% PFA. Next, retinas were dissected, flat mounted, and post-fixed for 1 h in 4% PFA. Brains were dissected and post-fixed overnight in 4% PFA. For BDNF IHC, perfusion was performed using 4% phosphate buffered PFA containing 0.4% parabenzoquinone (pBQ, Sigma-Aldrich, MO, USA), followed by brain post-fixation for 2 h in the same solution. The SC brain region was sectioned into 50 μm coronal sections using the Micron H650 vibratome (Thermo Scientific, MA, USA).

#### Immunohistochemistry on brain sections

For general IHC, free floating coronal SC sections were rinsed in PBS containing 0.1% Triton X-100 (Fisher Scientific, NY, USA) (PBST0.1) and incubated for 1 h in PBST0.1 containing 20% goat pre-immune serum (Invitrogen, CA, USA), followed by incubation with primary antibodies, diluted in PBST0.1 and 10% goat pre-immune serum (Table 1). After rinsing and incubation for 2 h with secondary antibody (Alexa Fluor-488/568-conjugated goat anti-rabbit and/or goat anti-mouse IgG, Invitrogen, CA, USA), diluted 1:200 in PBST0.1, sections were rinsed and cell nuclei were stained with 4’,6-diamidino-2-phenylindole (DAPI, 1 μg/ml in PBS, Dako, Denmark). Next, the tissue sections were mounted on gelatin-coated microscopy slides, and cover slipped with mowiol mounting medium (10% mowiol 4–88 (Sigma-Aldrich, MO, USA), 40% glycerol, 0.1% 1,4-diazabicyclo-[2,2,2]-octane in 0.2 M Tris-HCl [pH 8.5]).

**Table 1:** List of primary antibodies used for fluorescent IHC on coronal SC sections.

Primary antibody	Labels	Incubation time and dilution	Reference
Polyclonal rabbit anti-eGFP	eGFP	24 h, 1:10,000	In house made [39]
Monoclonal mouse anti-GFAP	(Reactive) astrocytes	24 h, 1:1,000	G3893, Sigma-Aldrich, MO, USA
Monoclonal mouse anti-NeuN	Neuronal nuclei	24 h, 1:200	MAB377, Millipore, Germany
Polyclonal rabbit anti-BDNF	Pro- and mBDNF	48 h, 1:500	sc-546, Santa Cruz Biotechnology, TX, USA

Key: IHC, immunohistochemistry; SC, superior colliculus; eGFP, enhanced green fluorescent protein; GFAP, glial fibrillary acidic protein; NeuN, neuronal nuclei; BDNF, brain-derived neurotrophic factor; mBDNF, mature form of brain-derived neurotrophic factor.

For NeuN IHC, before incubation with goat pre-immune serum, endogenous peroxidases were blocked via incubation in 0.3% H_2_O_2_ (in methanol) for 20 min, followed by thorough rinsing. After overnight primary antibody incubation, washing, and 45 min incubation with biotinylated goat anti-rabbit IgG (1:300 in PBST0.3, DAKO, Denmark), tyramid signal amplification via the TSA^™^ Cy3 System (NEL744001KT, Perkin Elmer, MA, USA) was used.

Immunofluorescent labeling was visualized using the FV1000-D laser confocal scanning microscope (Olympus Corporation, Japan), controlled by Fluoview 4.0 software.

#### RGC visualization and counting on whole-mount retinas

IHC labeling for Brn3a, a POU-domain transcription factor and reliable marker for RGCs[8, 27, 28], was performed on whole-mount retinas. Retinas were washed in PBS containing 0.5% Triton X-100 (PBST0.5) and frozen at -80°C for 15 min to enhance permeability. After rinsing in PBST0.5, retinas were incubated overnight with goat anti-Brn3a (1:500, sc-31984, Santa Cruz Biotechnology, TX, USA) diluted in PBS containing 2% Triton X-100 (PBST2.0) and 2% donkey pre-immune serum (Invitrogen, CA, USA). Subsequently, retinas were rinsed in PBST0.5 and incubated for 2 h with polyclonal Alexa Fluor-488-conjugated donkey anti-goat IgG (Invitrogen, CA, USA), diluted 1:500 in PBST2.0. Retinas were then rinsed in PBST0.5 and PBS before being mounted on glass slides with mowiol mounting medium. The stained retinal whole-mounts were imaged with the FV1000-D laser confocal scanning microscope (Olympus Corporation, Japan) controlled by Fluoview 4.0 software.

RGC density (number of RGCs/mm^2^) was calculated on images of the entire retinal whole-mount, using an in-house script developed for freely available Fiji software[40] (National Institute of Health, MD, USA) (Geeraerts *et al*., manuscript submitted to Exp Eye Res). Subsequently, RGC density data were transformed into pseudocolor isodensity maps to have a better view of the spatial differences in RGC density[31, 41, 42], with the pseudocolor scale representing low (dark blue) to high (red) RGC densities.

### *In vitro* secretion of vector-based BDNF by HEK cells

To verify cellular secretion of BDNF upon transduction with the BDNF-expressing vector, human embryonic kidney 293T (HEK293T) cells (American Type Culture Collection, VA, USA) were cultured in Dulbecco modified Eagle medium (DMEM, Invitrogen, CA, USA) supplemented with 8% fetal calf serum (Invitrogen, CA, USA) and gentamicin (Sabex, Canada), plated at 100,000 cells per well (24-well plates) in 500μl medium. HEK293T cells were transduced with either the rAAV2/1-CMV-BDNF or rAAV2/1-CMV-eGFP vector. After 48 h incubation with medium containing vector at 4.29E9 GC/ml, the cells were transferred to 6-well plates and medium was harvested 24 h and 72 h later. To quantify BDNF levels in the medium, the Mouse BDNF ELISA kit (KA0331, Abnova, Taiwan) was used according to the manufacturer’s guidelines. Analyses were performed with Skanit 3.1 software (Thermo Scientific, MA, USA).

To estimate transduction efficiency, eGFP vector-transduced cells were fixed for 15 min in 4% phosphate buffered PFA and incubated for 30 min with 1 μg/ml DAPI in PBS (Dako, Denmark) for nuclear staining. The DAPI and eGFP signals were visualized with the FV1000-D laser confocal scanning microscope (Olympus Corporation, Japan). The percentage of eGFP-expressing cells was calculated over 400 randomly chosen DAPI^+^ cells.

### *Ex vivo* validation of vector derived BDNF activity

#### Retinal explants

To validate the bio-activity of vector-derived BDNF, postnatal retinal explants were cultured in medium supplemented with conditioned medium derived from transduced HEK293T cells, and neurite outgrowth was studied. Retinal explants were isolated as previously described[43, 44]. Briefly, 750 μm explants were punched from dissected retinas from postnatal mouse pups (P3) and cultured in poly-L-lysin and laminin-coated wells (NunclonTM delta surface, Thermo Scientific, Rochester, NY, USA) for 3 d. Explants were cultured in Neurobasal^™^-A medium (Invitrogen, CA, USA), containing 0.4% methylcellulose (Sigma-Aldrich, MO, USA), 1% penicillin/streptomycin, 0.2% fungizone, 0.5% L-glutamine and 2% B-27 supplement for retina (all from Invitrogen, CA, USA). The explant culture medium was supplemented with 1) conditioned medium from BDNF vector-transduced HEK293T cells, to a final concentration of 50 ng/ml BDNF, 2) conditioned medium from eGFP vector-transduced HEK293T cells, or 3) with recombinant BDNF in a final concentration of 50ng/ml (Peprotech Laboratories, UK). Every day, half of the culture medium was replaced with fresh medium, containing the same concentration of compound or vehicle.

#### Immunohistochemistry on retinal explants and neurite outgrowth analysis

Explants were fixed in 4% phosphate buffered PFA and neurite outgrowth was visualized by immunostaining for β-tubulin using the monoclonal mouse anti-β-tubulin III antibody (1:500, T8660 clone SDL.3D10, Sigma-Aldrich, MO, USA), as described[43, 44]. β-tubulin III stained explants were imaged by the FV1000-D laser confocal scanning microscope controlled by Fluoview 4.0 software (Olympus Corporation, Japan) and analyzed using Axiovision software (Zeiss, Germany). Automated analysis of axonal outgrowth was performed by measuring the neurite outgrowth area, as previously described[43, 44].

### Statistics

All data are presented as mean ± SEM. Outliers were determined via a Grubbs test and excluded from further analyses. A normal distribution was verified using the Kolmogorov-Smirnov test and parallel equal variance between groups was tested. If the test requirements were fulfilled, a parametrical ANOVA was used, followed by bonferroni *post hoc* tests. When criteria for parametric statistics were not fulfilled, a non-parametrical Kruskal-Wallis analysis with a Mann-Whitney U rank sum test for pairwise comparison of independent samples was applied. Additionally, Student’s t-test for pairwise comparison was used. For all tests, a probability level (α-level was set to 0.05) of <0.05 was accepted as statistically significant (* p<0.05, * p<0.01, *** p<0.001). All statistical analyses were performed using GraphPad Prism S5 (GraphPad Software, CA, USA).

## Results

### RGC degeneration profiles in the mouse ONC and OHT models

Animal models of glaucoma are essential to elucidate the underlying pathophysiological mechanisms and to develop therapeutic strategies to halt or even reverse disease progression[32]. Therefore, our first goal was to outline RGC degeneration profiles in two commonly used experimental mouse models of glaucoma, the ONC and LP-induced OHT model, using Brn3a IHC at several time points post-injury.

**Fig 1:**
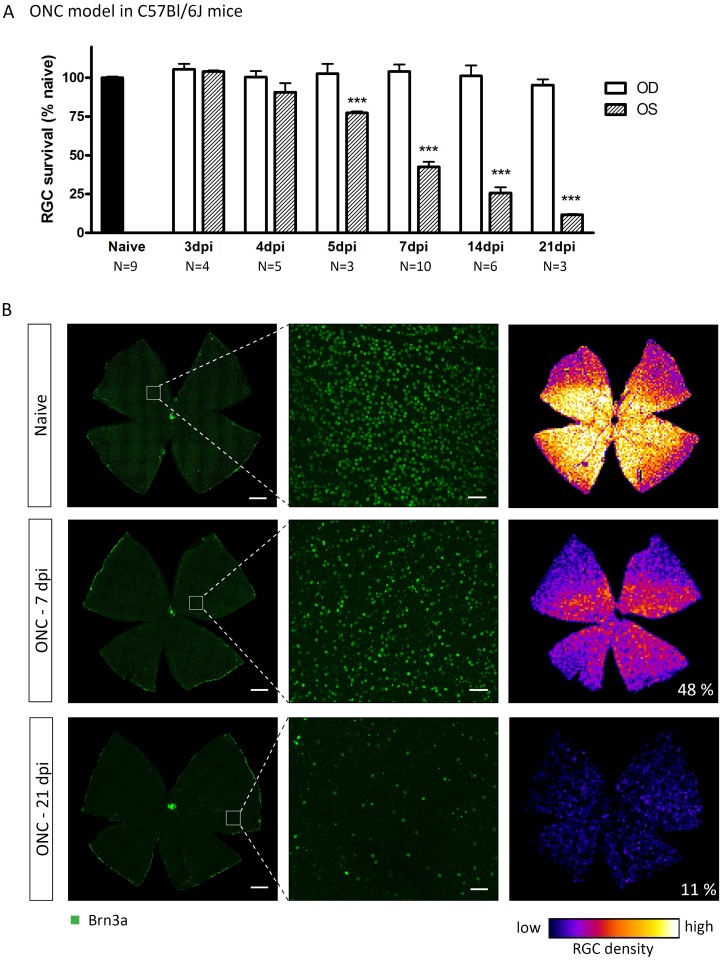
ONC yields progressive RGC degeneration, diffusely distributed throughout the retina. (A) ONC-induced RGC death in the ONC eye (OS) is progressive between 4 and 21 dpi. (B) Representative images of naive or injured retinas in which RGCs are visualized through Brn3a IHC. Right panels show isodensity maps with the numbers indicating percentages of surviving RGCs compared to naive. Scale bars left: 500 μm, middle: 50 μm. Key: ONC, optic nerve crush; RGC, retinal ganglion cell; IHC, immunohistochemistry; dpi, days post-injury; OS, *oculus sinister*; OD, *oculus dexter*.

The ONC induced with self-closing cross-action forceps resulted in a complete crush of the optic nerve and all RGC axons, as visualized by hematoxylin and eosin staining and RT97 IHC on optic nerve cross-sections, harvested at 1 h after ONC (data not shown). The onset of ONC-induced RGC death was situated between 4 dpi (3,102 ± 197.1 RGC/mm^2^, N = 5, p>0.05) and 5 dpi (2,550 ± 36.5 RGC/mm^2^, N = 3, p<0.001) (Fig 1A), while at 3 dpi, RGC densities (3,429 ± 20.8 RGC/mm^2^, N = 4) were similar to the naive condition (3,394 ± 29.4 RGC/mm^2^, N = 9). Between 5 and 21 dpi, RGC degeneration progressed, with RGC density measured at 7, 14, and 21 dpi being 1,392 ± 59.3 RGC/mm^2^ (N = 10), 756 ± 99.2 RGC/mm^2^ (N = 6), and 376 ± 12.7 RGC/mm^2^ (N = 3), respectively. At all time points, the contralateral eyes showed RGC densities comparable to naive retinas (on average 3,457 ± 67.8 RGC/mm^2^, N = 22). ONC-induced RGC degeneration was diffuse throughout the retina (Fig 1B).

**Fig 2:**
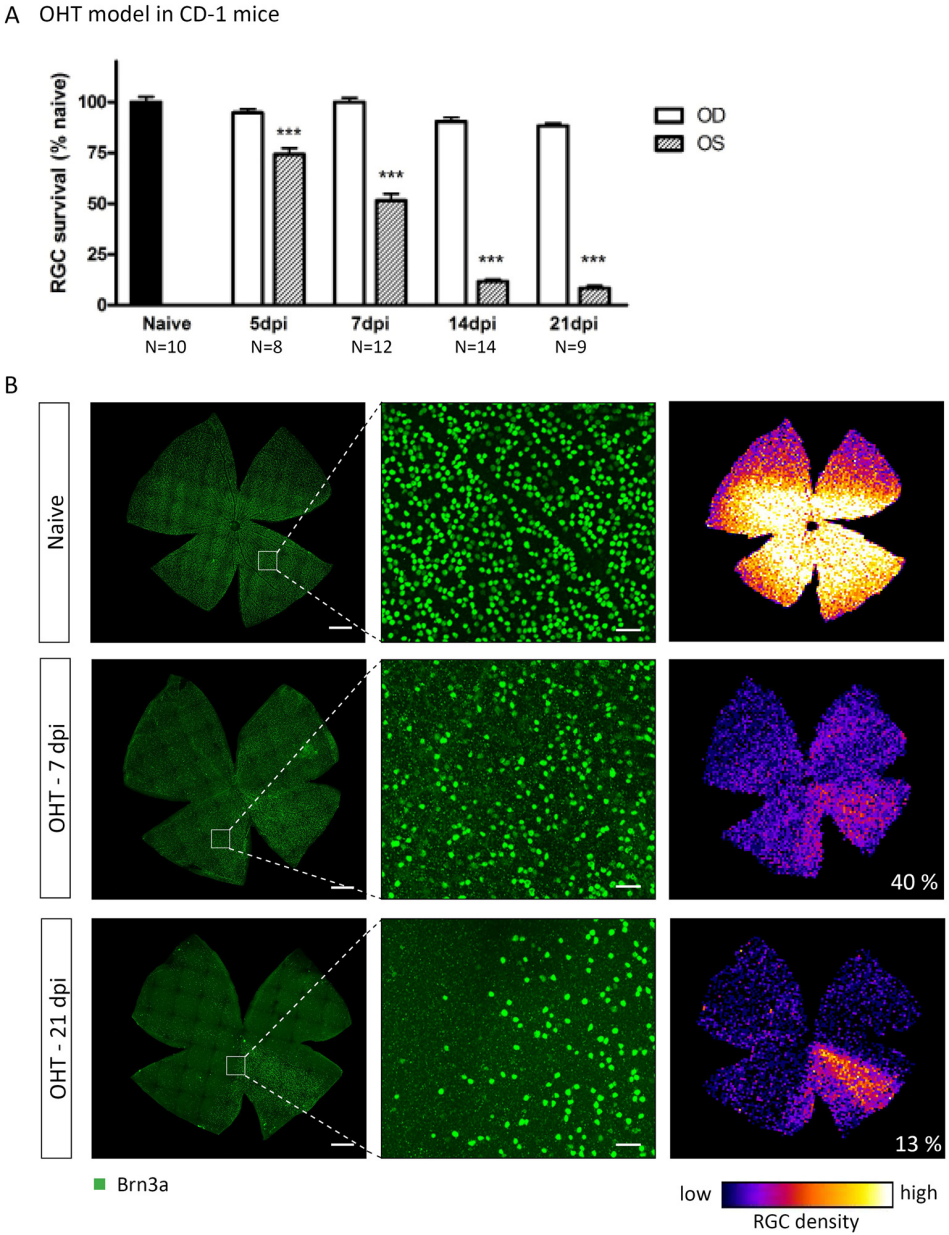
LP-induced OHT yields progressive diffuse and sectorial RGC degeneration. (A) OHT induces progressive RGC degeneration in the LP eye (OS), reaching final levels at 14 dpi. (B) Representative images of naive or injured retinas in which RGCs are visualized through Brn3a IHC. Right panels show isodensity map and the numbers indicate percentages of surviving RGCs compared to naive. Scale bars left: 500 μm, middle: 50 μm. Key: OHT, ocular hypertension; LP, laser photocoagulation; RGC, retinal ganglion cell; IHC, immunohistochemistry; dpi, days post-injury; OS, *oculus sinister*; OD, *oculus dexter*.

In the OHT model, from 24 h post-LP onwards, a significant increase in IOP was measured, resulting in an IOP of 41.9 ± 1.6 mmHg, compared to 12.8 ± 0.1 mmHg in the control eye. This OHT was sustained for at least 3 d, after which the IOP gradually declined to baseline levels by 7 d post-LP (data not shown), a pattern being in line with previous publications using this model[2, 30, 31]. RGC densities in the LP eyes were significantly reduced (p<0.001) at 5 dpi (3,010 ± 128.0 RGC/mm^2^, N = 8), 7 dpi (2,086 ± 140.9 RGC/mm^2^, N = 12), 14 dpi (470 ± 42.9 RGC/mm^2^, N = 14), and 21 dpi (337 ± 48.9 RGC/mm^2^, N = 9), compared to naive animals (4,065 ± 107.0 RGC/mm^2^, N = 10) (Fig 2A). RGCs died progressively between 5 and 14 dpi, while the RGC density remained stable thereafter. The contralateral eyes showed RGC densities comparable to naive retinas (on average 3,723 ± 51.4 RGC/mm^2^, N = 24). On top of diffusely distributed RGC death all over the retina, a pie-shaped sectorial degeneration pattern was observed (Fig 2B).

Thus, both the ONC and OHT models elicited progressive RGC degeneration. In both models, RGC loss occurred diffusely throughout the retina, however, only in the OHT model pie-shaped sectors of severe RGC death superposed the diffuse degeneration pattern.

### Expression of BDNF and TrkB in experimental glaucoma

In order to contribute to a better understanding of the role of neurotrophin signaling in glaucoma, the effect of experimental glaucoma on BDNF and TrkB levels in the glaucomatous retina and the SC contralateral to the experimental eye, was examined via ELISA for total BDNF levels and western blotting for TrkB-FL and TrkB-T1.

**Fig 3:**
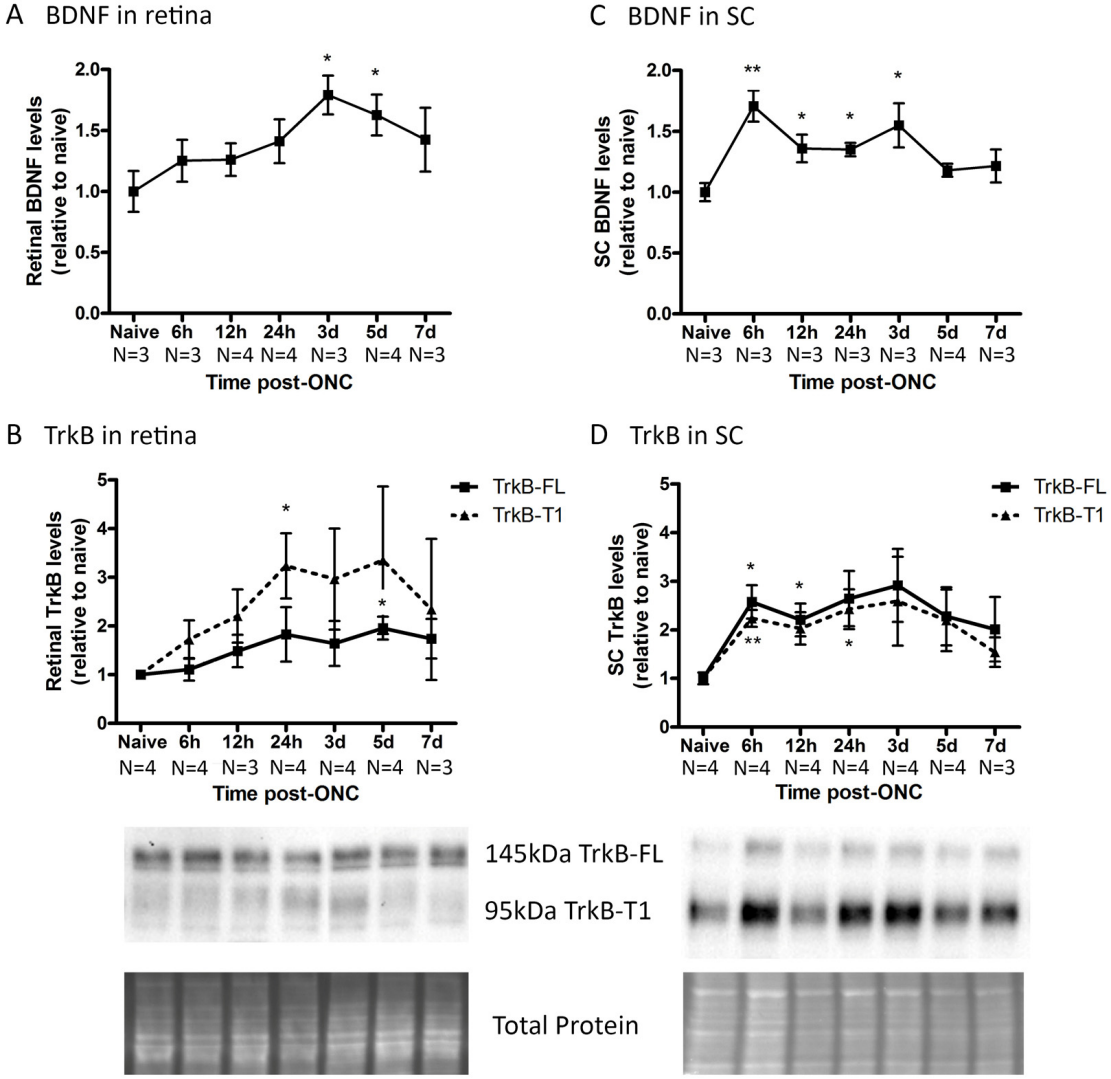
Endogenous BDNF and TrkB levels in retina and SC at various time points after ONC. (A) In the retina, the total BDNF levels, analyzed via ELISA, rise upon ONC, reaching a peak at 3 to 5 d, attenuating afterwards. (B) The retinal TrkB-FL levels only slightly increase in the ONC model, while TrkB-T1 inclines more, reaching peak levels between 24 h and 5 d post-ONC. (C) In the SC, total BDNF concentrations are enhanced as soon as 6 h post-ONC and slowly decrease to baseline levels thereafter. (D) Collicular TrkB-FL and TrkB-T1 levels follow a very similar increase-decrease pattern after ONC induction, with the turning point situated around 3 d post-ONC. Key: BDNF, brain-derived neurotrophic factor; TrkB, tropomyosin receptor kinase B; TrkB-FL, TrkB full-length form; TrkB-T1, TrkB truncated form 1; SC, superior colliculus; ONC, optic nerve crush.

In the ONC model, total BDNF levels in the retina increased marginally from 6 h post-ONC onwards, reaching significance at 3 dpi (+79 ± 15%, N = 3) to 5 dpi (+62 ± 16%, N = 4), and attenuating afterwards (Fig 3A). The retinal TrkB-FL levels increased slightly upon ONC (Fig 3B), with a maximum and the only significant difference at 5 dpi (+95 ± 23%, N = 4). TrkB-T1, in contrast, showed a larger increase, reaching peak levels between 24 h (+223 ± 66%, N = 3) and 5 d (+235 ± 151%, N = 4) post-ONC, however only the 24 h value differed significantly from the naive condition. In the SC, total BDNF levels (Fig 3C) were significantly enhanced as soon as 6 h post-ONC (+70 ± 12%, N = 4) and slowly decreased to baseline levels thereafter, remaining significantly different from naive until 3 dpi (+55 ± 18%, N = 4). Collicular TrkB-FL and TrkB-T1 levels (Fig 3D) followed a very similar pattern after ONC induction, increasing from 6 hpi onwards (TrkB-FL +158 ± 34%, TrkB-T1 +123 ± 17%, N = 4) with the turning point situated around 3 d post-ONC (TrkB-FL +192 ± 74%, TrkB-T1 +159 ± 91%, N = 4). Strikingly, in the retina TrkB-FL is the most abundant form, while in the SC, TrkB-T1 is more represented.

**Fig 4:**
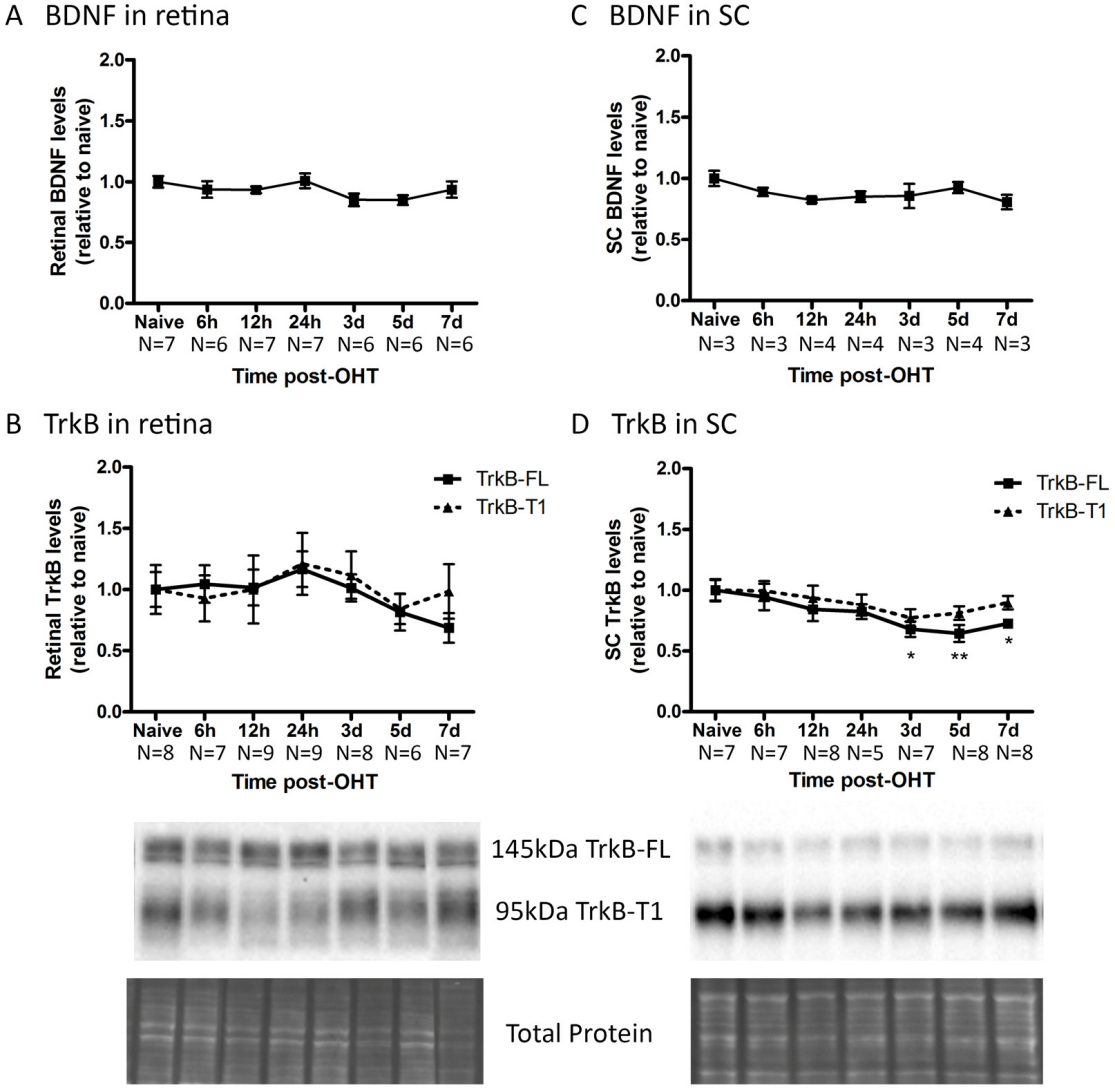
Endogenous BDNF and TrkB levels in retina and SC at various time points after OHT induction. (A) In the retina, the total BDNF levels do not change by OHT induction. (B) The retinal levels of TrkB-FL and TrkB-T1 do not show significant alterations in the OHT model. (C) In the SC, the total BDNF levels are not altered by OHT induction. (D) Collicular TrkB-FL and TrkB-T1 levels follow a very similar, slightly decreasing pattern after OHT induction. Key: BDNF, brain-derived neurotrophic factor; TrkB, tropomyosin receptor kinase B; TrkB-FL, TrkB full-length form; TrkB-T1, TrkB truncated form 1; SC, superior colliculus; OHT, ocular hypertension.

For the OHT model, patterns of BDNF and TrkB levels in the retina and SC were different from the ONC model. In the retina of OHT eyes, no changes in the total BDNF level, neither in the TrkB-FL and TrkB-T1 levels, could be detected, in comparison to naive eyes (Fig 4A and 4B). In the SC, total BDNF levels were not noticeably altered by OHT induction either (Fig 4C). However, OHT induction did induce a slight but significant decrease in collicular TrkB-FL levels (Fig 4D), with the lowest point situated at 5 dpi (-36 ± 6%, N = 8). TrkB-T1 levels followed a similar decreasing pattern in the OHT model, however, this decline did not reach significance.

Overall, retinal and collicular BDNF and TrkB levels were differentially altered after experimentally induced glaucoma in the ONC and OHT models.

### Tackling glaucoma from within the brain: a viral vector based approach

According to the neurotrophin deprivation hypothesis, disruption of retrograde delivery of trophic support is one of the main triggers that induces apoptotic signaling in the RGCs in glaucoma[5, 45]. With the cellular response to neurotrophin-receptor interaction being suggested to be different depending on the neuronal compartment from where the signal is initiated (*i*.*e*. in the cell body, in the axon terminals, or on the dendrites)[18, 23], it seems plausible to hypothesize that delaying or preventing RGC death could be better achieved by interfering with their target areas. We intended to deliver a proof-of-concept for this hypothesis, by studying the effect of local neurotrophin delivery in the SC on the RGC survival in different glaucoma models. To induce a chronic neurotrophin upregulation in the SC, viral vector technology was used.

#### Validation of viral vector based BDNF overexpression *in vivo*

**Fig 5:**
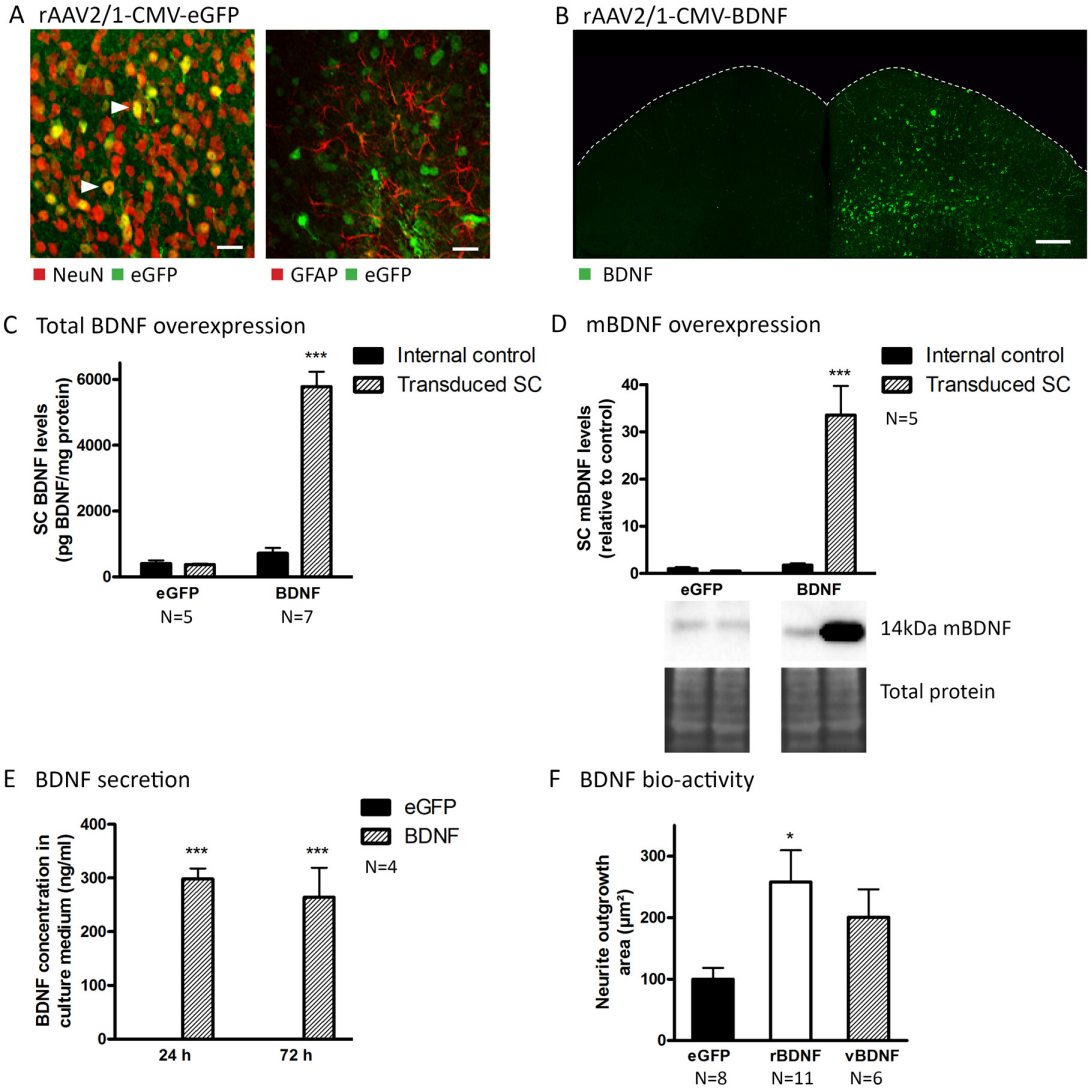
Validation of expression, secretion, and activity of viral vector-derived BDNF. (A) The rAAV2/1-CMV-eGFP vector almost exclusively induces eGFP expression in neurons (NeuN) and not in glial cells (GFAP). Filled arrowheads point towards double-labeled cells. Scale bars: 20 μm. (B) Unilateral rAAV2/1-CMV-BDNF vector-induced BDNF upregulation is visualized through IHC. Scale bar: 200 μm. (C) Total BDNF levels in BDNF vector-transduced SC are sharply enhanced compared to non-transduced SC in the same animals (internal control) and to both SCi of eGFP vector-transduced mice, quantified via ELISA. (D) Western blot analyses show an upregulation of mBDNF levels in BDNF vector-transduced SC compared to internal controls and to eGFP vector- transduced animals. (E) In contrast to HEK293T cells transduced with the eGFP control vector, HEK293T cells transduced with BDNF vector secrete large amounts of BDNF in the culture medium, as quantified by ELISA on medium harvested 24 h and 72 h post-transduction. (F) Retinal explants cultured in medium supplemented with recombinant BDNF (rBDNF) or vector-derived BDNF (vBDNF) show enhanced neurite outgrowth areas compared to control explants (eGFP). N = number of explants, derived from three mice. Key: BDNF, brain-derived neurotrophic factor; mBDNF, mature form of BDNF; eGFP, enhanced green fluorescent protein; DAPI, 4’,6-diamidino-2-phenylindole; SC, superior colliculus; rAAV2/1, recombinant adeno-associated viral vector serotype 2/1; CMV, cytomegalovirus promoter; IHC, immunohistochemistry; ELISA, enzyme-linked immunosorbent assay; ONC, optic nerve crush; OHT, ocular hypertension; dpi, days post-injury; HEK293T, Human embryonic kidney cell line.

Based on the fact that the applicability of different viral vector systems depends on the experimental setup, tissue, and species[46, 47], we first defined the optimal vector system for local delivery to the mouse SC[48]. Thereto, the viral vector of choice was determined by comparing the induced glial response, transduction efficiency, cell tropism, and retrograde transduction capacity of different rAAV2/x serotypes upon delivery to the SC. Since our goal was to study the effect of enhanced neurotrophin release within the SC on RGC survival in glaucoma models, it seemed essential to pick a viral vector that effectively targets collicular cells, but does not result in retrograde transduction of retinal cells. As vector rAAV2/1 was found to elicit low astroglial reactivity and to exhibit high transduction efficiency in mouse SC neurons (Fig 5A) without retrogradely targeting the retina, this vector was chosen for all further experiments. For a more detailed description (comparison of the viral serotypes tested) of these results we refer to S1 Fig.

Next, the rAAV2/1-CMV-BDNF vector, developed to induce collicular neurotrophin overexpression, was validated *in vivo*. Unilateral delivery of the rAAV2/1-CMV-BDNF vector into the mouse SC resulted in extensive upregulation of BDNF expression, visualized through IHC (Fig 5B) at two weeks post-injection, as compared to the non-injected hemisphere. In addition, total BDNF levels, quantified using ELISA at 18 d post vector delivery (Fig 5C) were highly augmented in BDNF vector-transduced SC (5,780 ± 453 pg BDNF/mg protein, N = 7) as compared to both non-transduced SCi in the same animals (716 ± 162 pg BDNF/mg protein, N = 7) or to eGFP vector-transduced SCi (407 ± 91 pg BDNF/mg protein, N = 5). Since pro- and mature forms of BDNF partially induce divergent signaling cascades, it was important to verify whether transgenically expressed pro-BDNF was transformed into mBDNF. This was validated through western blotting, in which a clear upregulation of the 14 kDa mBDNF band was identified in transduced SC samples derived from BDNF vector-injected mice, as compared to both the internal control (+567 ± 168.4%, N = 5) and eGFP vector-transduced SC of external control animals (+462 ± 116.4%, N = 5) (Fig 5D). Thus, mBDNF was clearly upregulated in the mouse SC upon vector delivery *in vivo*.

#### *In vitro/ex vivo* validation of vector-derived BDNF activity

The characteristics of vector-derived BDNF were investigated in more detail *in vitro/ex vivo*. To verify whether transgenically expressed BDNF could be secreted by transduced cells, BDNF concentrations were measured in medium harvested from cultured HEK293T cells, transduced at a success rate of 81.2% with either the BDNF- or eGFP-expressing viral vector. As expected, BDNF levels were much higher (***p<0.001, N = 4) in medium harvested from BDNF vector-transduced cells, both at 24 h (298.45 ± 19.51 ng/ml) and at 72 h (264.03 ± 55.24 ng/ml) post-transduction, as compared to medium harvested from cells transduced with the control vector at 24 h (0.01 ± 0.00 ng/ml) and 72 h (0.01 ± 0.00 ng/ml) post-transduction (Fig 5E). Thus, viral vector-derived BDNF is secreted by transduced HEK293T cells *in vitro*. Next, using the well-established retinal explant model, bio-activity of the vector-derived BDNF was verified. Explants incubated with medium supplemented with recombinant BDNF (rBDNF, N = 11) or with conditioned medium from BDNF vector-transduced HEK293T cells (vBDNF, N = 6) showed enhanced neurite outgrowth compared to control explants, which were cultured in medium supplemented with equal amounts of conditioned medium from eGFP vector-transduced HEK293T cells (eGFP, N = 8) (Fig 5F). Thus, viral vector-derived BDNF was shown to be bio-active.

#### Intracollicular BDNF delivery is not neuroprotective to RGCs in the ONC and OHT model

In the following experiments, we envisioned to counteract glaucomatous RGC degeneration via brain-directed treatment, by stimulating the pro-survival colliculoretinal signaling. Thereto, the validated rAAV2/1-CMV-BDNF vector was used to upregulate BDNF in the mouse SC in an attempt to induce long-distance neurotrophic protective effects on the RGCs in both models of experimental glaucoma.

**Fig 6:**
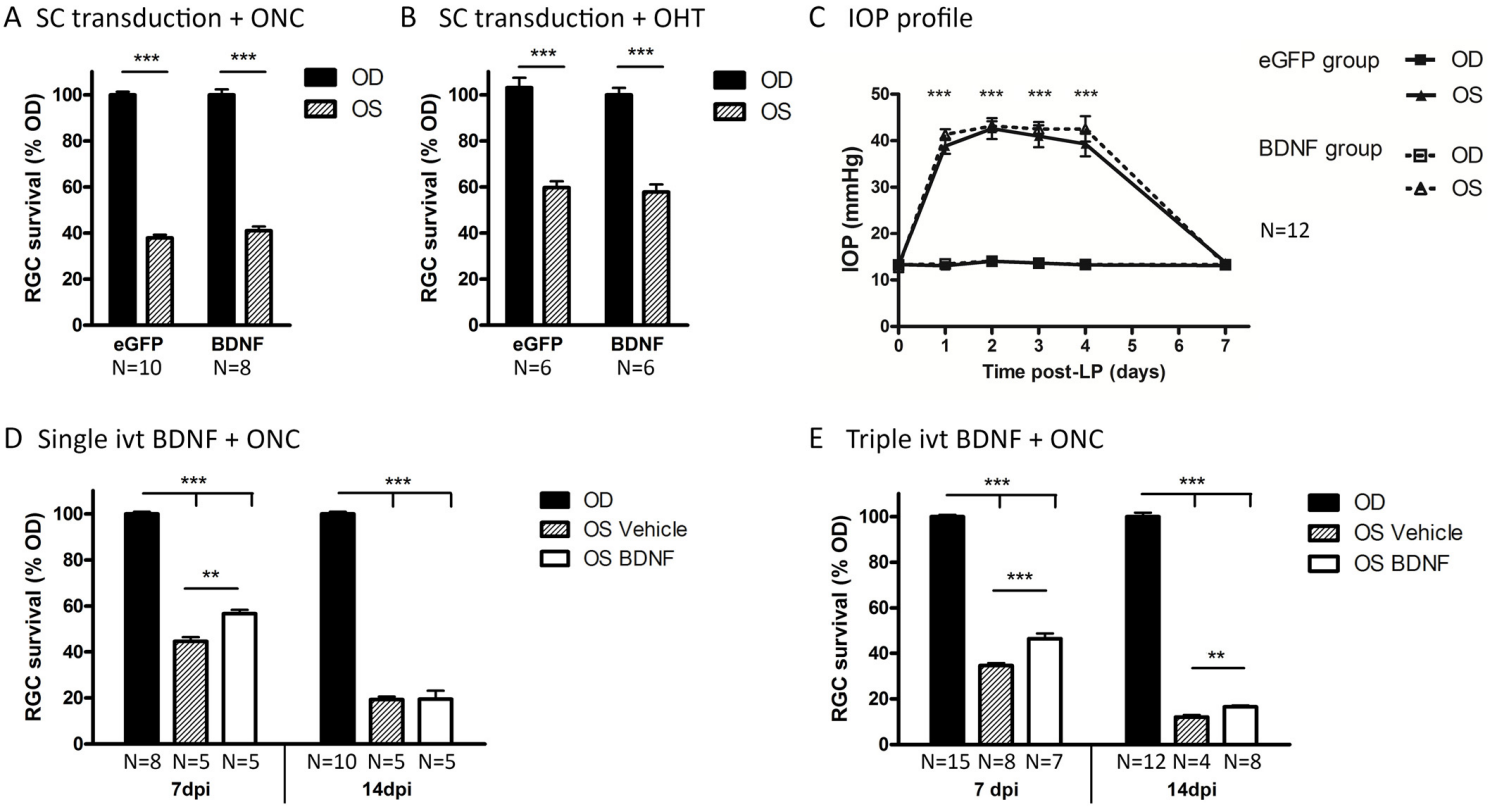
Glaucomatous RGC death is counteracted by ivt BDNF administration but not by vector-mediated BDNF upregulation in the SC. (A) At 7 d post-ONC, no difference is detected in RGC survival between groups with eGFP or BDNF transgene expression in the SC. (B) At 7 d post-OHT induction, collicular eGFP and BDNF transgene expressing mice show a similar degree of RGC degeneration. (C) IOP elevation in both eGFP and BDNF transgene expressing groups follow a similar pattern. Key: ivt, intravitreal; RGC, retinal ganglion cell; SC, superior colliculus; OD, *oculus dexter*; OS, *oculus sinister*; BDNF, brain-derived neurotrophic factor; eGFP, enhanced green fluorescent protein; LP, laser photocoagulation; days post-injury. D. One single ivt injection of BDNF at the time of ONC, leads to a temporarily enhanced RGC survival of 10% at 7 dpi, but not at 14 dpi. E. Repeated ivt BDNF administration at 0, 3, and 5 dpi could not ameliorate the survival effect obtained with one single injection at 7 dpi. However, triple BDNF treatment at 0, 5, and 10 dpi did result in a prolonged increase in RGC survival of 4.5% at 14 dpi.

Two weeks after intracollicular vector delivery to induce chronic BDNF or eGFP expression, mice received unilateral ONC or OHT induction and were sacrificed 7 d later. First, BDNF and eGFP expression was validated via IHC in three randomly chosen animals of each group. For each of these mice, a robust transgene expression was observed throughout the SC (data not shown, similar to results of vector validation shown in Fig 5B). Next, RGC survival was evaluated. Within the ONC model, RGC counts were not found to be different between treatment groups (Fig 6A), with RGC densities of 1,326 ± 54.7 RGCs/mm^2^ and 1,170 ± 89.3 RGCs/mm^2^ measured in ONC eyes of the BDNF vector-transduced group (N = 8) and the eGFP group (N = 10), respectively. Also, for the OHT model, in which IOP patterns were very similar between groups (Fig 6C), no differences were detected in RGC survival rates between BDNF and eGFP groups (Fig 6B). Measured RGC densities in the hypertensive eye were 2,386 ± 214.7 RGCs/mm^2^ for the BDNF group (N = 6) and 2,473 ± 192.2 RGCs/mm^2^ for the eGFP group (N = 6).

Thus, despite a clear BDNF upregulation in the SC, the viral vector-mediated brain treatment did not exert neuroprotective effects on RGCs in the present experimental glaucoma models.

### Explanation for the unexpected hurdles in the viral vector based treatment

#### Intravitreal BDNF administration is neuroprotective in the ONC model

We then sought for an explanation for these disappointing results. First, we verified whether the experimentally induced RGC degeneration in our models leaves a window to induce neuroprotection. Thereto, mice that were subjected to the ONC model received local BDNF treatment through ivt injection, based on previous reports[8–10, 14].

A single ivt administration of recombinant mBDNF at the time of ONC resulted in 10% neuroprotection at 7 d after ONC (Fig 6D). Brn3a^+^ RGC density was 2,125 ± 52.7 RGCs/mm^2^ and 1,658 ± 65.4 RGCs/mm^2^ in BDNF- and vehicle-treated ONC eyes, respectively (N = 4). However, the protective effect was transient, as at 14 dpi, there was no longer a difference in Brn3a^+^ RGC counts between BDNF- (700 ± 65.8 RGCs/mm^2^) and vehicle-treated (710 ± 93.2 RGCs/mm^2^) ONC eyes (N = 5).

Strikingly, triple BDNF administration at 0, 3, and 5 dpi was not found to enhance the neuroprotective effect seen at 7 dpi after a single dose (Fig 6E). Using this repetitive treatment paradigm, again, only a 10% reduction in RGC death was detected in BDNF-treated ONC eyes (1,785 ± 38.7 RGCs/mm^2^, N = 7) at 7 dpi, as compared to vehicle-injected eyes (1,393 ± 41.9 RGCs/mm^2^, N = 8). However, ivt BDNF injections at 0, 5, and 10 dpi did slightly prolong the neuroprotective effect, with 4.5% enhanced survival in the ONC eyes of the BDNF (N = 8) versus vehicle (N = 4) groups at 14 dpi. RGC densities in ONC eyes were 532 ± 20.2 RGCs/mm^2^ in BDNF-treated groups versus 389 ± 28.4 RGCs/mm^2^ in the vehicle group.

Thus, our results confirmed the temporary RGC protective effect of ivt delivered recombinant BDNF in the ONC model. Despite its ability to prevent RGC degeneration in the short-term, the long-term effectiveness of ivt BDNF administration was limited, even when provided as multiple injections.

#### Viral vector-induced collicular BDNF overexpression results in neurotrophin desensitization

**Fig 7:**
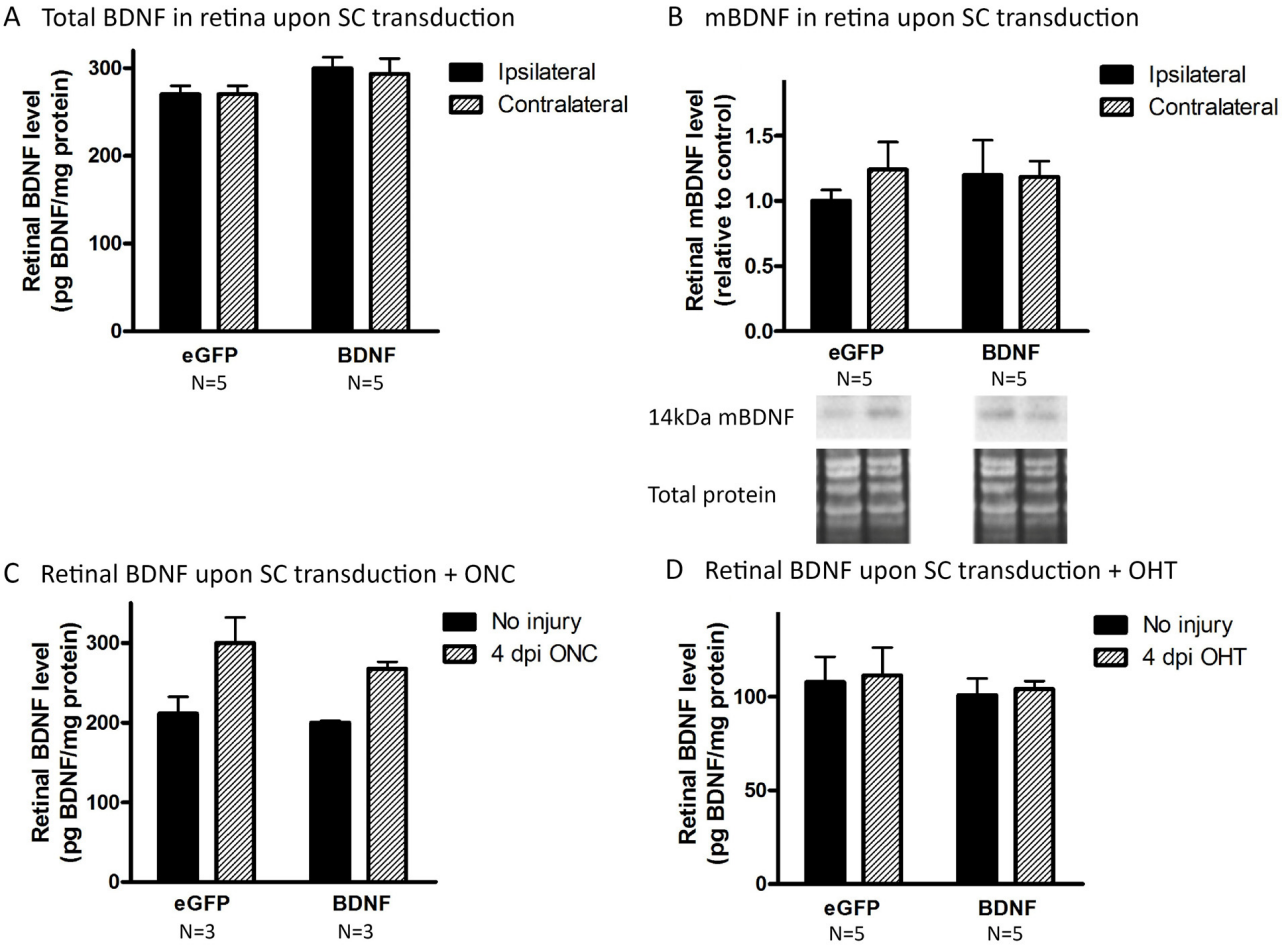
Viral vector-derived BDNF in the SC is not transported towards the retina. (A) Unilateral vector-based BDNF upregulation in the SC does not lead to changes in total BDNF levels in the retina, ipsilateral nor contralateral to the transduced SC, as measured via ELISA. (B) Western blot analysis for detection of mBDNF does not show any difference between retina ipsilateral nor contralateral to the BDNF or eGFP vector-transduced SC. (C) Total retinal BDNF levels in ONC or non-ONC eyes, contralateral to the transduced SC, are similar in mice injected with rAAV2/1-CMV-BDNF or rAAV2/1-CMV-eGFP. (D) Similar results as shown in C. were obtained using OHT induction instead of ONC. Key: BDNF, brain-derived neurotrophic factor; mBDNF, mature form of BDNF; ELISA, enzyme-linked immunosorbent assay; eGFP, enhanced green fluorescent protein; ONC, optic nerve crush; OHT, ocular hypertension; dpi, days post-injury.

**Fig 8:**
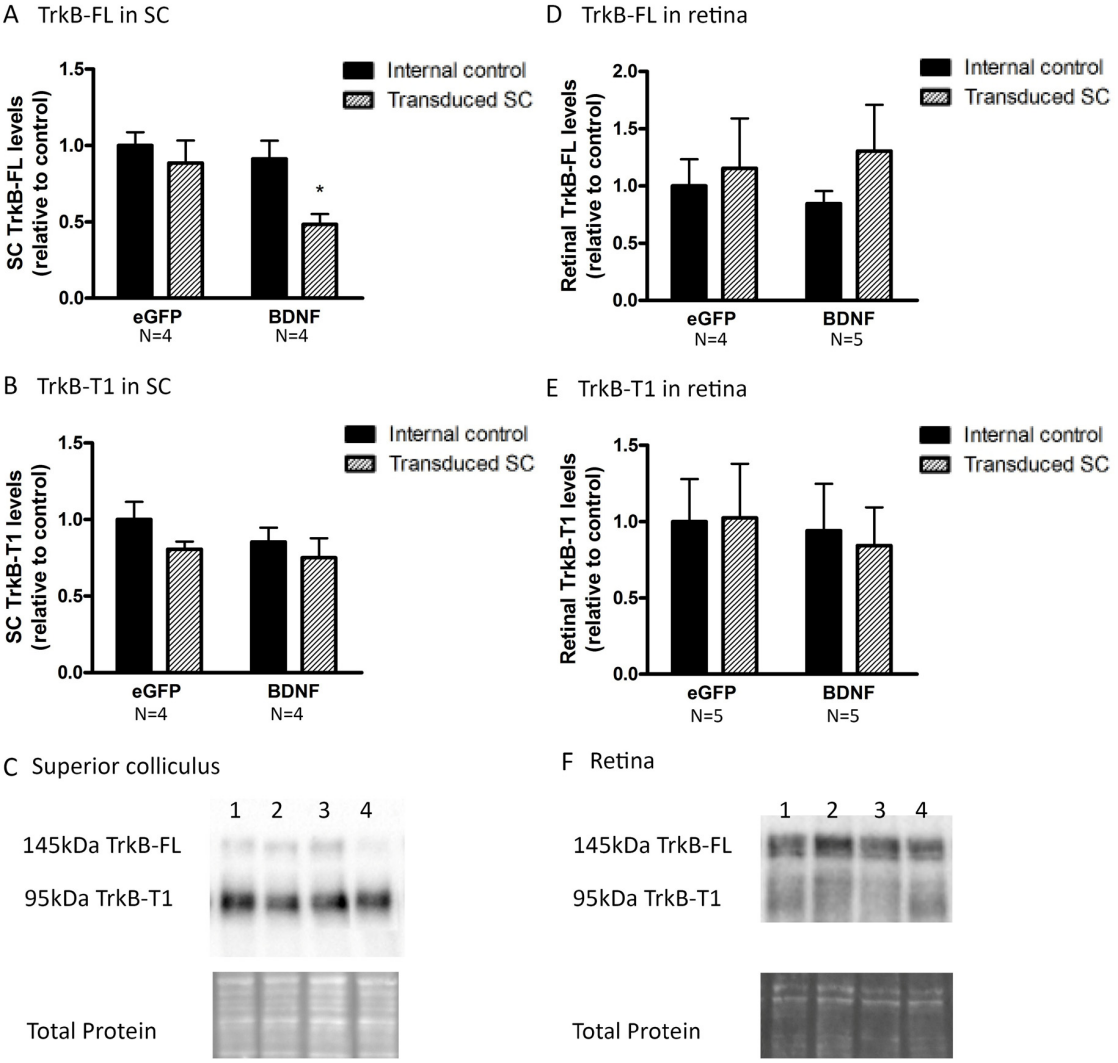
Collicular and retinal TrkB levels upon unilateral viral vector delivery to the SC. (A) Western blot analysis shows reduced TrkB-FL levels in BDNF vector-transduced SC compared to both internal controls and eGFP vector-transduced animals. (B) Collicular TrkB-T1 levels are not influenced by unilateral SC vector transduction. (C) Western blot for TrkB-FL and TrkB-T1 in the SC 18 d after viral vector delivery showing representative bands for the graphs in A and B, with 1) internal control SC of eGFP vector injected mice, 2) SC transduced with eGFP vector, 3) internal control SC of BDNF vector-transduced mice, 4) BDNF vector-injected SC. (D, E) Upon unilateral SC transduction with eGFP or BDNF vector, no differences in full-length (D) or truncated (E) forms of TrkB are detected in retinas ipsilateral or contralateral to the transduced SC. F. TrkB-FL and TrkB-T1 western blot detection in the retina 18 d after viral vector delivery, representative bands for the graphs in C and D, with 1) internal control retina ipsilateral to eGFP vector-injected SC, 2) retina contralateral to the SC transduced with eGFP vector, 3) internal control retina ipsilateral to BDNF vector-transduced SC, 4) retina contralateral to BDNF vector-injected SC. Key: SC, superior colliculus; BDNF, brain-derived neurotrophic factor; TrkB, tropomyosin receptor kinase B; TrkB-FL, TrkB full-length form; TrkB-T1, TrkB truncated form 1, eGFP, enhanced green fluorescent protein.

Then why did the delivery of BDNF to the SC not have a similar neuroprotective outcome? To be able to support RGC survival, BDNF, released and activated within the SC, needs to reach the retina via retrograde transport through the RGC axons. Unfortunately, both ELISA and western blot analyses of retinal samples (N = 5), dissected at 18 d post collicular BDNF vector injection, revealed no change in total and mBDNF levels in the retina despite a clear BDNF overexpression in the SC (Fig 7A and 7B). Possibly, RGCs do not retrogradely transport the extra BDNF unless additional trophic support is needed, e.g. to counteract injury. Therefore, retinal BDNF measurements were repeated in animals, in which viral vector injection was combined with experimentally induced glaucoma. Thereto, mice were subjected to ONC (N = 3) and OHT (N = 5) at 14 d post vector injection, and samples were processed 4 d post glaucoma induction. Based on the fact that ELISA is more sensitive than western blotting, ELISA was the method of choice here. Unfortunately, retinal BDNF levels remained unaltered upon vector-mediated collicular BDNF overexpression (Fig 7C and 7D). Our data confirmed the upregulation of retinal BDNF upon ONC described above, but no additional effect of SC transduction was seen. Overall, we were not able to establish any effect on retinal BDNF levels upon BDNF upregulation in the SC, in non-injured nor injured eyes. One possible explanation for this lack of retrograde transport is an abnormal expression or distribution of the TrkB receptor. Indeed, for its colliculoretinal transport, BDNF needs to bind to its receptor in order to be internalized into a transport vesicle. Therefore, we determined TrkB-FL and TrkB-T1 levels in both SC and retina upon vector injection. Importantly, at 18 d post vector delivery, vector-based enhancement of BDNF levels in the SC led to a reduction of TrkB-FL levels in the BDNF vector-transduced SC (-47 ± 7.3% compared to internal control, N = 4, Fig 8A), while TrkB-T1 levels did not change (N = 4, Fig 8B). Viral vector-derived BDNF did not affect retinal TrkB-FL (N_BDNF_ = 4, N_eGFP_ = 5, Fig 8D) and TrkB-T1 levels (N = 5, Fig 8E).

Thus, our data indicated that in response to vector-based BDNF upregulation in the SC, TrkB-FL levels were downregulated in the transduced SC, thereby possibly reducing complex formation between BDNF and TrkB-FL and hence colliculoretinal transport of BDNF.

## Discussion

The main objective of the performed experiments was to study the contribution of BDNF, the most commonly studied neurotrophin, to glaucoma disease progression, using two experimental mouse glaucoma models, commonly applied to mimic glaucoma pathology.

### RGC degeneration patterns in the ONC and OHT model

We first characterized the RGC degeneration pattern for both models. ONC-triggered retinal neurodegeneration had an onset between 4 and 5 dpi, progressively evolving towards a survival ratio of 12% at 21 dpi. RGC loss elicited by ONC appeared diffuse and affected the whole retina, without any evidence of a sectorial pattern, which is in line with literature[28, 49]. Different RGC degeneration rates upon ONC have been reported by others, reasoned to be caused by variations in experimental procedures, such as the distance between the crush site and the globe[49], and the duration and force of the crush[32], or the method of RGC visualization and quantification[50].

Within the OHT model, characterized by a temporary elevation of the IOP, RGC loss occurred diffusely throughout the retina with pie-shaped sectors of severe RGC death superposing the diffuse degeneration pattern, as clearly demonstrated by the isodensity maps. This pattern reflects a typical glaucomatous retinal degeneration pattern described in human patients[51] and several OHT-related animal models[2, 31, 52–54]. Although IOP elevation was acute and transient, which may be considered a disadvantage when keeping in mind the chronic nature of human glaucoma, OHT-induced degeneration continued after IOP had normalized. Indeed, a decrease in RGC density was apparent with increasing time post-LP. Other studies, using the same model in CD-1 mice, reported Brn3a-based survival rates that are in accordance with our results[30, 31]. Of note, care must be taken when comparing various reports describing survival rates upon OHT-induced by LP of the perilimbal and episcleral veins in mice, since the use of different mouse strains[54] or various methods to label RGCs[52], might lead to discrepancies in the reported degeneration profiles.

### Endogenous expression of BDNF and TrkB in experimental glaucoma

#### Experimental glaucoma affects BDNF and TrkB levels in the retina

**Fig 9:**
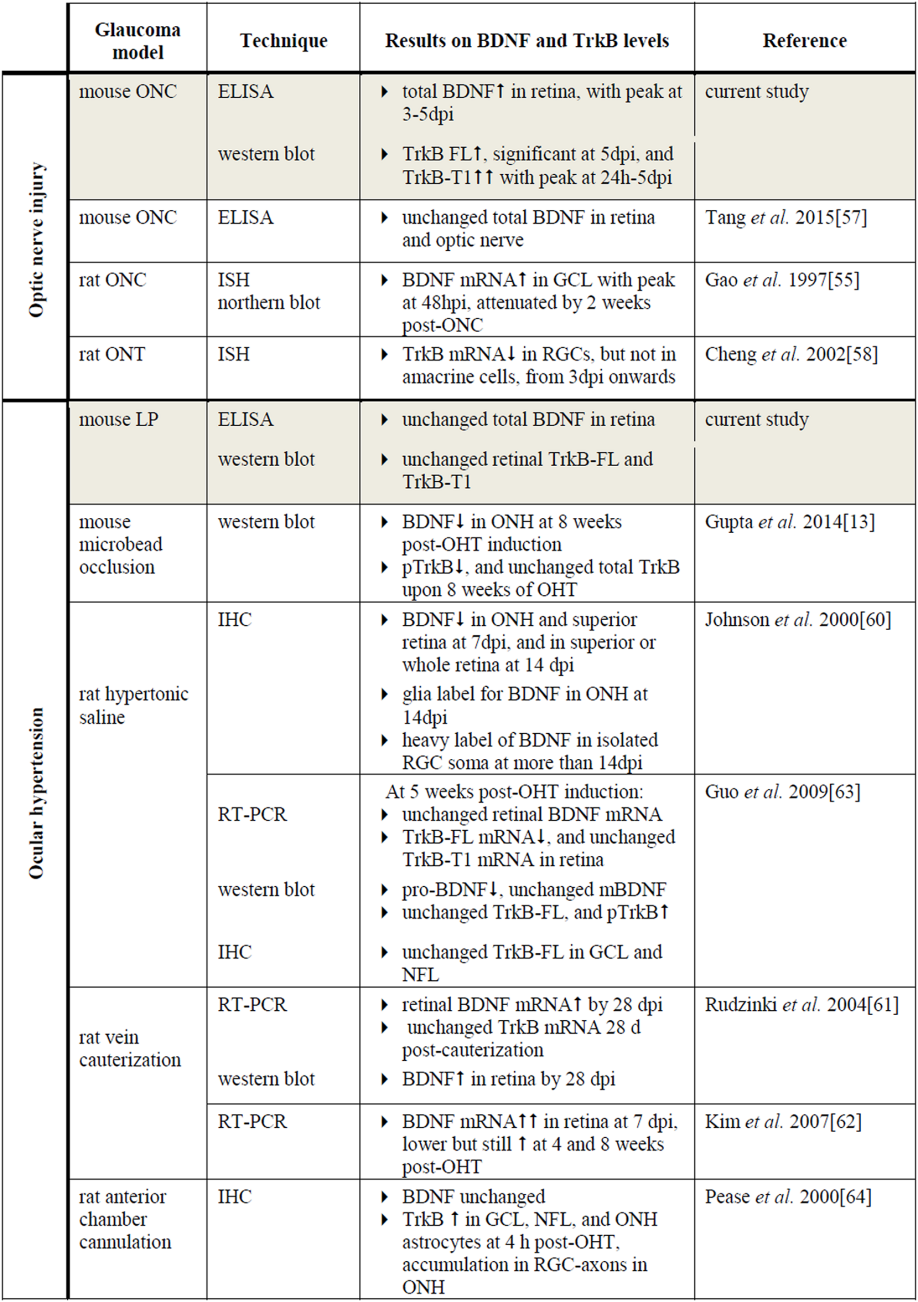
Literature overview on retinal BDNF and TrkB expression in murine optic nerve injury and OHT models. Key: BDNF, brain-derived neurotrophic factor; mBDNF, mature form of BDNF; TrkB, tropomyosin receptor kinase B; TrkB-FL, TrkB full-length form; TrkB-T1, TrkB truncated form 1; pTrkB, phosphorylated TrkB; ONC, optic nerve crush; LP, laser photocoagulation; OHT, ocular hypertension; ONH, optic nerve head; GCL, ganglion cell layer; NLF, nerve fiber layer; ELISA, enzyme-linked immunosorbent assay; ISH, in situ hybridization; IHC, immunohistochemistry; RT-PCR; reverse transcription polymerase chain reaction; hpi, hours post-injury; dpi, days post-injury.

The current study, investigating levels of BDNF together with its main receptor TrkB in the retina and SC in two mouse models of experimentally induced glaucoma, firstly demonstrated that retinal BDNF levels were elevated after ONC (Fig 9). ONC induces immediate axonal damage that results in blockage of the retrograde delivery of neurotrophins from the brain. Rapid upregulation of endogenous BDNF expression in the retina may thus act as a natural protection mechanism to overcome neurotrophin transport deficits[6, 55]. In line with our results, temporary upregulation of BDNF[55] and also of downstream actors within neurotrophin-induced pathways[56], have been previously described in the ONC model; however, others reported no effect of ONC on retinal BDNF concentrations[57]. Furthermore, the retinal levels of both the TrkB-FL and TrkB-T1 isoforms increased upon ONC, with TrkB-T1 showing the most pronounced increase. This might seem contrary to the study of Cheng *et al*., describing reduced TrkB levels in rat RGCs after optic nerve transection (ONT)[58]. However, it is important to note that the results of the present work reflect findings from the whole retina, and that responses unique to RGCs may be diluted by contributions from other TrkB-expressing retinal cells, e.g. Müller glia[59].

Although both BDNF and TrkB concentrations increased in the retina after ONC, this did not protect RGCs from dying, possibly because the TrkB upregulating effect was the highest for the TrkB-T1 isoform. The expected neuroprotective role of the extra BDNF might be attenuated because of sequestration by TrkB-T1 expressing glia[22] or because of a shift towards TrkB-T1 in the TrkB ratio in RGCs.

In contrast to ONC, BDNF and TrkB levels in the retina were not affected by OHT in our study (Fig 9). Although OHT has also been described to negatively influence RGC axonal transport, leading to neurotrophin deprivation[6], this effect is believed to be less direct compared to ONC. The observed differences in endogenous expression of BDNF and TrkB within the 2 glaucoma models then indeed hint to a (partial) preservation of axonal transport in the OHT model as compared to ONC at the included time points. Of note, while the ELISA data revealed stable total BDNF levels, changes in the pro-/mBDNF ratio cannot be excluded.

Literature describing the effect of OHT on retinal BDNF signaling is confusing and far from complete, with various studies, using a number of rodent OHT models, showing assorted effects of elevated IOP on BDNF and TrkB mRNA and protein levels in the retina and ONH (Fig 9)[13, 60–63]. These discrepancies in the existing reports are probably the resultant of differences in how OHT is induced and the level of the resultant IOP elevation, as well as of the techniques applied for sample processing and subsequent mRNA and protein detection.

#### Experimental glaucoma affects BDNF and TrkB levels in the superior colliculus

**Fig 10:**
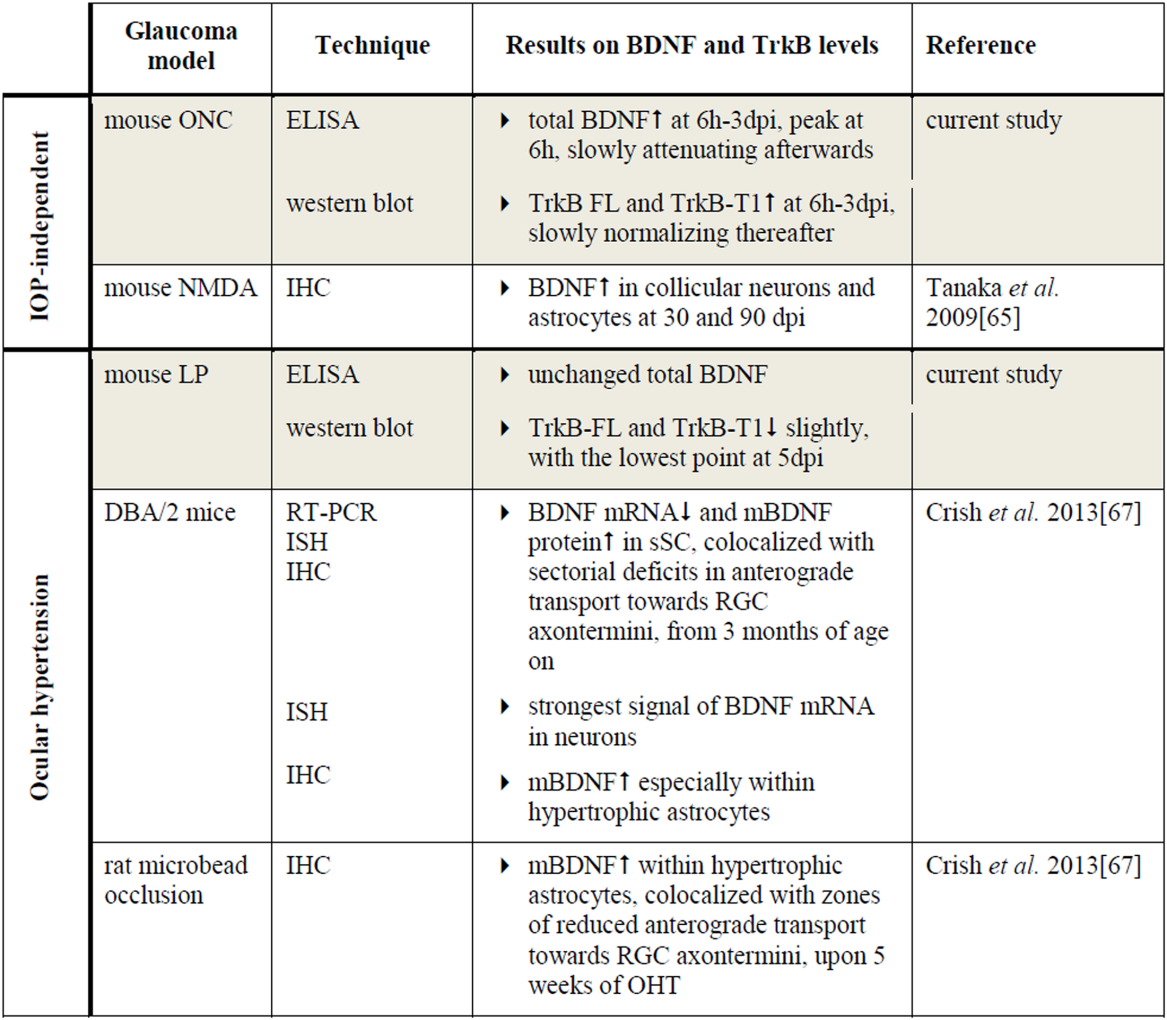
Literature overview on collicular BDNF and TrkB expression in murine models of glaucoma. Key: BDNF, brain-derived neurotrophic factor; mBDNF, mature form of BDNF; TrkB, tropomyosin receptor kinase B; TrkB-FL, TrkB full-length form; TrkB-T1, TrkB truncated form 1; ONC, optic nerve crush; LP, laser photocoagulation; sSC, the superficial layers of the superior colliculus; NMDA, N-methyl-D-aspartate; ELISA, enzyme-linked immunosorbent assay; ISH, in situ hybridization; IHC, immunohistochemistry; RT-PCR; reverse transcription polymerase chain reaction; hpi, hours post-injury; dpi, days post-injury.

In the SC, total BDNF was significantly enhanced as soon as 6 h post-ONC, slowly decreasing to baseline levels afterwards (Fig 10). Collicular TrkB-FL and TrkB-T1 levels followed a parallel increase-decrease pattern after ONC. To date, the link between ONC and BDNF/TrkB expression in the retinal target areas has not yet been elucidated. However, since ONC directly obstructs the retinal output, producing a quick drop in RGC-derived input into the visual brain centers, the observed responses in the SC soon after ONC were not entirely unpredicted. Upregulation of BDNF and TrkB in retinal target areas might in fact be an attempt to prevent post-synaptic degeneration. Similarly, in the N-methyl-D-aspartate (NMDA) model, BDNF upregulation in the dLGN and the SC has also been associated with RGC degeneration[65, 66].

In the OHT model, no significant effect on the BDNF concentration in the SC was measured (Fig 10). In comparison, in DBA/2 mice, pre-degenerative transport deficits were found to be accompanied by a decrement in BDNF mRNA expression in the retinorecipient SC, while conversely, mBDNF protein IHC signal increased, an observation that was confirmed in the microbead-based OHT model[67]. The fact that no differences were measured in the collicular BDNF levels within our study might be related to the model and the time points analyzed. However, the additional possibility remains that a redistribution of BDNF occurs, changing its location and/or the pro-/mBDNF ratio without modifying the total concentrations. Nevertheless, our results did illustrate a slight reduction in TrkB-FL and TrkB-T1. Quite possibly, this reduction stems from diminished anterograde TrkB delivery from the cell soma in the retina towards the axon terminals in the SC or downregulation of TrkB expression by collicular cells. Alternatively, the functional and structural degeneration of the RGC axon terminals might well lead to reduced TrkB levels in the SC, since RGC synaptic terminals in the SC are affected as soon as one week post-OHT induction[30].

Thus, BDNF-TrkB signaling is regulated by spatial and temporal changes in protein localization, gene expression, the mBDNF/pro-BDNF ratio, the TrkB-FL/TrkB-T1 ratio, and downstream signaling molecules. Defining a specific role for BDNF signaling within glaucoma pathology remains difficult, as various studies, using a number of animal glaucoma models, have culminated in a variety of unique results[63], the present work included.

### Intravitreal delivery of BDNF delays glaucomatous RGC degeneration while collicular BDNF upregulation does not

Based on the neurotrophin deprivation hypothesis, neurotrophins gained wide acceptance as suitable candidates for neuroprotective drug usage. Indeed, upon experimentally induced glaucoma, RGCs have been described to survive longer after ivt administration of various neurotrophic factors[9, 10, 68]. Amongst these, BDNF has been consistently shown as one of the most powerful[7, 9, 69], though only delivering a transient neuroprotection[9, 45]. Our data confirmed this temporary RGC protective effect of ivt delivery of recombinant BDNF in the ONC model. The neuroprotective power of ivt BDNF may be caused by enhanced BDNF-TrkB signaling in RGCs, resulting in the activation of pro-survival signaling pathways and the suppression of apoptotic mechanisms[26, 70]. Yet, ivt BDNF might also affect RGC survival indirectly via stimulation of Müller cells, to secrete other neuron supporting compounds[71]. Retinal TrkB levels were not downregulated upon ONC, as discussed above, and so ivt administered BDNF could indeed provide neuroprotection by promoting TrkB signaling in RGCs or glia. However, the TrkB-FL/TrkB-T1 ratio shifted slightly towards the T1 isoform, of which the exact role is not fully understood. Although initially defined as a dominant-negative receptor, the truncated receptor also seems to fulfill other, possibly even survival promoting, functions[22]. Therefore, it is hard to draw strong conclusions on how exactly ivt BDNF delivery promotes RGC survival and why this effect is transient. Presumably, the latter is partly due to limited supply when administered ivt, but also to downregulation of the relevant receptors and desensitization of the signal transduction pathways[7, 26, 72]. The decrease in responsiveness to BDNF most likely stems from a downregulation in TrkB receptor availability, brought about by decreased TrkB expression, internalization of the BDNF-bound receptors with reduced replacement in the cell membrane, or competitive interactions with truncated TrkB receptors[26]. Accordingly, combining ivt BDNF injections with transduction of RGCs with the TrkB gene, notably enhanced the duration of ivt BDNF-induced neuroprotection after optic nerve axotomy in rats[58].

Since local BDNF administration to the eye appears insufficient for sustained RGC survival, we hypothesized that treatment at the level of extraretinal neurotrophin sources in the brain might be favorable. Indeed, Weber *et al*. have demonstrated that combined application of BDNF to the eye and the central visual pathway enhanced RGC survival and function relative to treatment of the eye alone, in an ONC model in cats[7, 26]. Based on this finding, we intended to deliver BDNF to the RGCs from a natural extraretinal source, the SC, in order to induce RGC neuroprotection in murine glaucoma models. Unfortunately, although viral vector-mediated BDNF upregulation in the mouse SC was validated, the attempts to protect RGCs upon ONC or OHT through collicular vector treatment were unsuccessful. Nonetheless, vector-derived BDNF was shown to be secreted by transduced cells *in vitro*, and to be transformed into the mature form *in vivo*. In addition, viral vector-derived BDNF was demonstrated to be bio-active, since it stimulated neurite outgrowth in an *ex vivo* retinal explant culture. Notably, as viral vector-based BDNF upregulation in the SC elicited effects on the availability of its receptor (discussed in detail below), our findings clearly indicate that transgenically expressed BDNF was secreted into the extracellular space by transduced collicular neurons *in vivo* as well.

In line with our data, based on unpublished results showing that treatment of the visual cortex alone was insufficient to provide RGC neuroprotection, Weber *et al*. postulated that the enhanced survival achieved in their dual treatment paradigm was the result of the contribution from both the eye and visual brain center treatment[7, 26]. They hypothesized that direct treatment of the eye is necessary to sustain RGCs during the immediate post-injury period, in order to counter the initial insult and short-term degenerative effects, whereas treatment of the brain center provides more long-term stability. However, some additional issues need to be taken into account. Indeed, more detailed investigation of responses to viral vector-mediated BDNF overexpression revealed that upregulation of BDNF in the SC did not result in any enhancement of BDNF levels in the retina. Thus, transgenically expressed BDNF did not seem to be retrogradely transported by RGC axons. This was the case in both healthy conditions and after ONC- or OHT-induced injury. Perhaps, if sufficient levels are available, RGCs do not alter their normal transport rate. Of note, we cannot entirely exclude that some technical aspects might have hampered the retrograde delivery of BDNF. Indeed, at the moment of ONC, axonal transport is abruptly blocked in all RGC axons. Furthermore, although axonal damage has been postulated to be rather indirectly in the OHT model[30, 31], the strong IOP elevation seen in this study may possibly result in rather fast diminishment of the retrograde axonal transport[64]. However, also in healthy conditions, viral-vector derived BDNF was not transported from the SC towards the retina. This observation is in contrast with previous studies, demonstrating retrograde transport of collicular-injected tagged BDNF in rats, which is diminished and accompanied by accumulation of TrkB receptor in the ONH upon OHT induction[64]. Of note, the more chronic feature of our setup, using viral vector based long-term overexpression of BDNF, in comparison to the acute delivery of tagged BDNF with sacrifice 4 h later, might explain the different results. Indeed, our unfortunate finding might well be attributed to the demonstrated downregulation of collicular TrkB-FL levels as a response to chronic BDNF upregulation and secretion. Potentially, this downregulatory effect may be explained by a reduction in the delivery of TrkB from the retina to the RGC axon terminals of the target area. Assuming this downregulation of TrkB-FL is due to reduced availability of TrkB-FL on the RGC axon terminals, the viral vector-derived BDNF will not be taken up at the RGC synapses and thus will not be transported towards the retina. In keeping with the observation that collicular TrkB-T1 levels did not change upon vector delivery, another possibility to explain the lack of retrograde transport of vector-derived BDNF remains. Indeed, it is plausible that the viral vector-derived BDNF is sequestered by glia in an effort to maintain extracellular BDNF levels within normal ranges. In our opinion, downregulation of the TrkB-FL expression at the RGC axon terminals seems the most reasonable explanation for the absence of retrograde transport of the vector-derived BDNF. This is in agreement with previous findings in the retina, whereby downregulation of TrkB has been described upon ivt injection of recombinant BDNF[7, 58, 72]. Thus, additional research should consider restoration of trophic responsiveness by increasing the number of TrkB-FL receptors present on the RGC terminals surface, e.g. by overexpression[58, 73] or by enhancing their recruitment to the plasma membrane[74].

## Conclusion

The temporal course of RGC degeneration was characterized in two mouse models of experimentally induced glaucoma, *i*.*e*. ONC and LP-induced OHT. Furthermore, endogenous BDNF and TrkB expression patterns in the retina and SC were shown to be differentially affected upon ONC or OHT induction. Using the ONC model, the neuroprotective capacity of ivt BDNF administration was confirmed. Unfortunately, viral vector-induced overexpression of BDNF in the SC did not result in enhanced RGC survival in none of the two glaucoma models. These negative results seemed mainly attributed to reduced neurotrophin responsiveness as a response to vector-mediated BDNF overexpression and secretion. Overall, our data provide important information for the design of additional research on the neurotrophin-deprivation-hypothesis in glaucoma.

## Supporting Information

S1 Fig Induced astroglial response, transduction efficiency, and retrograde transduction capacity of different viral vectors upon delivery into the mouse SC. (A) Two weeks after intracollicular delivery of different viral vectors (rAAV2/1, 2/7, 2/8, and 2/9), expressing the eGFP gene under control of the general CMV promoter, astroglial reactivity, defined by upregulated GFAP labeling, remains locally around the injection site (± 300 μm) for each vector and the difference between rAAV vector-injected SC and sham controls is limited. Scale bars: 100 μm. (B) Unilateral transduction with rAAV2/1, rAAV2/7, rAAV2/8, and rAAV2/9, visualized through CMV promoter-regulated eGFP expression, spreads throughout the entire SC in anteroposterior and lateromedial direction. 200 μm spaced serial sections between Bregma -3.30 mm and -4.50 mm are depicted. Scale bars: 200 μm. (C) In contrast to rAAV2/1, unilateral injection of rAAV2/7, rAAV2/8, and rAAV2/9 vectors into the mouse SC results in retrograde transduction and eGFP expression in RGCs, identified through Brn3a IHC, in the eye contralateral to the injected SC. Arrowheads point towards double-labeled cells, showing green cytoplasm and a red nucleus. Scale bars: 20 μm. Key: GFAP, glial fibrillary acidic protein; IHC, immunohistochemistry; SC, superior colliculus; RGC; retinal ganglion cell; eGFP, enhanced green fluorescent protein; rAAV2/x, recombinant adeno-associated viral vector serotype 2/x; CMV, cytomegalovirus promoter.
(TIF)

S1 Text Supplementary methods: determination of the best vector type for transduction of the mouse SC. (PDF)
