# Supplementary figures and images for: Tackling Glaucoma from within the Brain: An Unfortunate Interplay of BDNF and TrkB
Source: PLoS One. 2015 Nov 11;10(11):e0142067. doi: 10.1371/journal.pone.0142067 (PMC4641732; doi:10.1371/journal.pone.0142067)

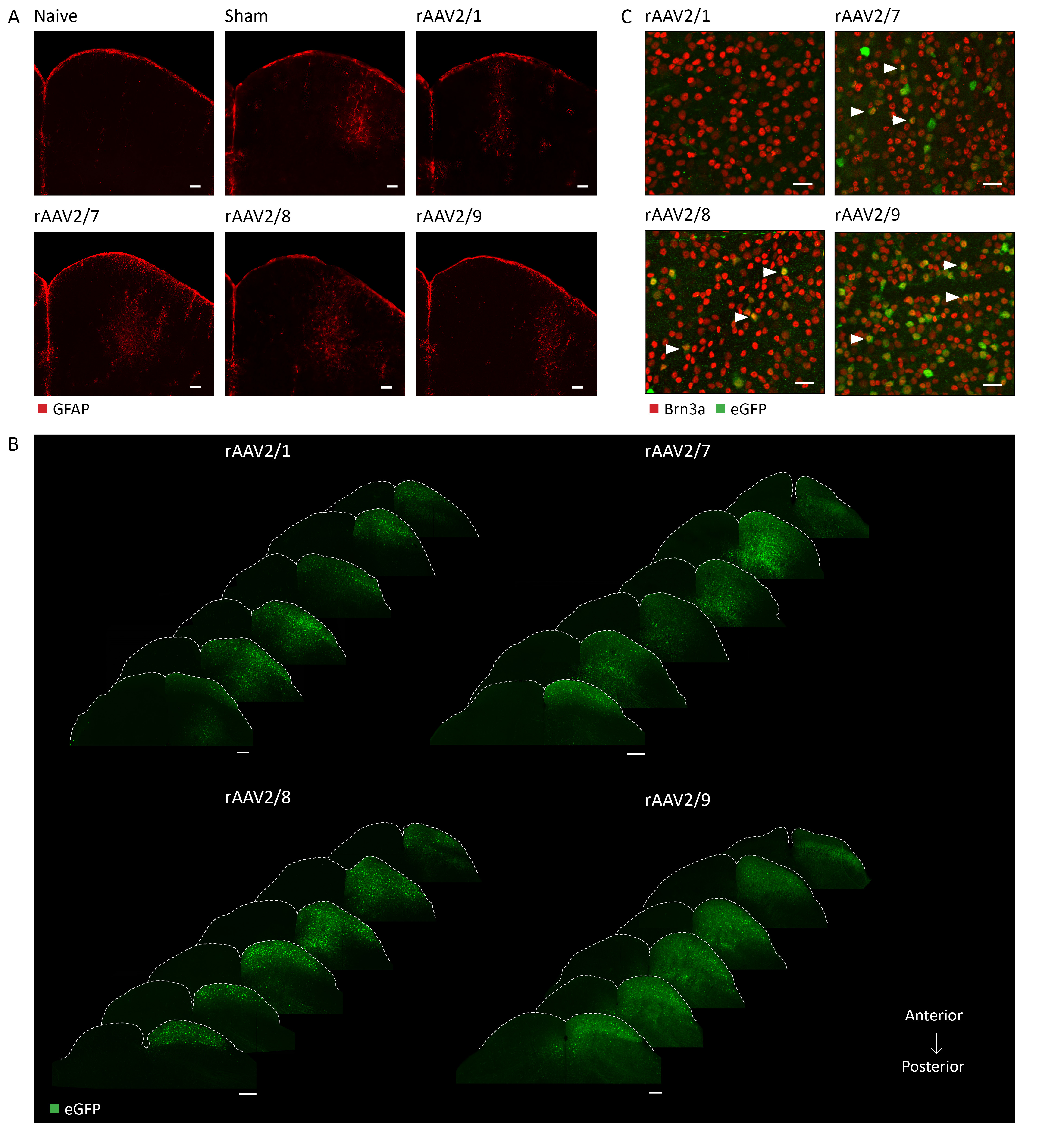

Supplement: S1 Fig — (A) Two weeks after intracollicular delivery of different viral vectors (rAAV2/1, 2/7, 2/8, and 2/9), expressing the eGFP gene under control of the general CMV promoter, astroglial reactivity, defined by upregulated GFAP labeling, remains locally around the injection site (± 300 μm) for each vector and the difference between rAAV vector-injected SC and sham controls is limited. Scale bars: 100 μm. (B) Unilateral transduction with rAAV2/1, rAAV2/7, rAAV2/8, and rAAV2/9, visualized through CMV promoter-regulated eGFP expression, spreads throughout the entire SC in anteroposterior and lateromedial direction. 200 μm spaced serial sections between Bregma -3.30 mm and -4.50 mm are depicted. Scale bars: 200 μm. (C) In contrast to rAAV2/1, unilateral injection of rAAV2/7, rAAV2/8, and rAAV2/9 vectors into the mouse SC results in retrograde transduction and eGFP expression in RGCs, identified through Brn3a IHC, in the eye contralateral to the injected SC. Arrowheads point towards double-labeled cells, showing green cytoplasm and a red nucleus. Scale bars: 20 μm. Key: GFAP, glial fibrillary acidic protein; IHC, immunohistochemistry; SC, superior colliculus; RGC; retinal ganglion cell; eGFP, enhanced green fluorescent protein; rAAV2/x, recombinant adeno-associated viral vector serotype 2/x; CMV, cytomegalovirus promoter. (TIF) [file pone.0142067.s001.tif]
