# Supplementary material for: Tackling Glaucoma from within the Brain: An Unfortunate Interplay of BDNF and TrkB
Source: PLoS One. 2015 Nov 11;10(11):e0142067. doi: 10.1371/journal.pone.0142067 (PMC4641732; doi:10.1371/journal.pone.0142067)
Supplement: S1 Text — (PDF) [file pone.0142067.s002.pdf]

S1 Text.

Supplementary methods: determination of the vector type for localized transduction of the mouse SC.

The viral vectors used to determine the serotype for optimal transduction of the mouse SC, were produced by the Viral Vector Core of KU Leuven, as described previously by Van der Perren *et al.*[1]. Four rAAV vectors, expressing the enhanced green fluorescent protein (eGFP) gene under control of the general cytomegalovirus (CMV) promoter, were tested for transduction in the SC. The vectors all contained the inverted terminal repeats (ITRs) of AAV2, but were cross packaged in the capsids of AAV1, AAV7, AAV8, or AAV9, resulting in four different vector serotypes: rAAV2/1 (1.10E12 GC/ml), rAAV2/7 (1.90E12 GC/ml), rAAV2/8 (2.61E12 GC/ml), and rAAV2/9 (3.20E12 GC/ml).

200 nl of the different rAAV vectors was stereotactically injected into the right superior colliculus (SC) to study expression patterns of the eGFP reporter gene (N=4). For sham controls, 200 nl sterile PBS was injected (N=3).

Serial coronal SC sections were immunostained for eGFP to estimate transduction efficiency and for GFAP, to visualize elicited astroglial responses. Double staining for eGFP and GFAP, or eGFP and NeuN, allowed to distinguish between viral vector targeting of neurons and astrocytes. IHC protocols are described in the main text.

In order to examine possible retrograde transduction of the retina after viral vector injection into the SC, double IHC labeling for eGFP and Brn3a was performed on whole-mount retinas, following the Brn3a protocol described in the main text, with addition of the polyclonal primary antibody rabbit anti-eGFP (1:10,000, provided by Prof. V. Baekelandt[2]) and the polyclonal secondary donkey Alexa Fluor-conjugated anti-rabbit IgG (Invitrogen, CA, USA).

1. Van der Perren A, Toelen J, Carlon M, Van den Haute C, Coun F, Heeman B, et al. (2011) Efficient and stable transduction of dopaminergic neurons in rat substantia nigra by rAAV 2/1, 2/2, 2/5, 2/6.2, 2/7, 2/8 and 2/9. Gene Ther. 18: 517-27.
2. Baekelandt V, Eggermont K, Michiels M, Nuttin B, Debyser Z (2003) Optimized lentiviral vector production and purification procedure prevents immune response after transduction of mouse brain. Gene Ther. 10: 1933-40.
